# RNAi Screen of Endoplasmic Reticulum–Associated Host Factors Reveals a Role for IRE1α in Supporting *Brucella* Replication

**DOI:** 10.1371/journal.ppat.1000110

**Published:** 2008-07-25

**Authors:** Qing-Ming Qin, Jianwu Pei, Veronica Ancona, Brian D. Shaw, Thomas A. Ficht, Paul de Figueiredo

**Affiliations:** 1 Department of Plant Pathology and Microbiology, Texas A&M University, College Station, Texas, United States of America; 2 Department of Veterinary Pathobiology, Texas A&M University, College Station, Texas, United States of America; 3 Faculty of Genetics, Texas A&M University, College Station, Texas, United States of America; 4 Professional Program in Biotechnology, Texas A&M University, College Station, Texas, United States of America; 5 Faculty of Molecular and Environmental Plant Systems, Texas A&M University, College Station, Texas, United States of America; Stanford University, United States of America

## Abstract

*Brucella* species are facultative intracellular bacterial pathogens that cause brucellosis, a global zoonosis of profound importance. Although recent studies have demonstrated that *Brucella* spp. replicate within an intracellular compartment that contains endoplasmic reticulum (ER) resident proteins, the molecular mechanisms by which the pathogen secures this replicative niche remain obscure. Here, we address this issue by exploiting *Drosophila* S2 cells and RNA interference (RNAi) technology to develop a genetically tractable system that recapitulates critical aspects of mammalian cell infection. After validating this system by demonstrating a shared requirement for phosphoinositide 3-kinase (PI3K) activities in supporting *Brucella* infection in both host cell systems, we performed an RNAi screen of 240 genes, including 110 ER-associated genes, for molecules that mediate bacterial interactions with the ER. We uncovered 52 evolutionarily conserved host factors that, when depleted, inhibited or increased *Brucella* infection. Strikingly, 29 of these factors had not been previously suggested to support bacterial infection of host cells. The most intriguing of these was inositol-requiring enzyme 1 (IRE1), a transmembrane kinase that regulates the eukaryotic unfolded protein response (UPR). We employed IRE1α^−/−^ murine embryonic fibroblasts (MEFs) to demonstrate a role for this protein in supporting *Brucella* infection of mammalian cells, and thereby, validated the utility of the *Drosophila* S2 cell system for uncovering novel *Brucella* host factors. Finally, we propose a model in which IRE1α, and other ER-associated genes uncovered in our screen, mediate *Brucella* replication by promoting autophagosome biogenesis.

## Author Summary

*Brucella* spp. are facultative intracellular pathogens that cause brucellosis in a broad range of hosts, including humans. *Brucella melitensis*, *B. abortus*, and *B. suis* are highly infectious and can be readily transmitted in aerosolized form, and a human vaccine against brucellosis is unavailable. Therefore, these pathogens are recognized as potential bioterror agents. Because genetic systems for studying host–*Brucella* interactions have been unavailable, little is known about the host factors that mediate infection. Here, we demonstrate that a *Drosophila* S2 cell system and RNA interference can be exploited to study the role that evolutionarily conserved *Brucella* host proteins play in these processes. We also show that this system provides for the identification and characterization of host factors that mediate *Brucella* interactions with the host cell endoplasmic reticulum. In fact, we identified 52 host factors that, when depleted, inhibited or increased *Brucella* infection. Among the identified *Brucella* host factors, 29 have not been previously shown to support bacterial infection. Finally, we demonstrate that the novel host factor inositol-requiring enzyme 1 (IRE1) and its mammalian ortholog (IRE1α) are required for *Brucella* infection of *Drosophila* S2 and mammalian cells, respectively. Therefore, this work contributes to our understanding of host factors mediating *Brucella* infection.

## Introduction

Infectious diseases caused by intracellular bacterial pathogens are responsible for an enormous amount of worldwide pain, suffering, and mortality. *Brucella* spp., for example, cause brucellosis, a global zoonosis of profound importance [1],[2]. *Brucella melitensis*, *B. abortus*, and *B. suis* are highly infectious and can be readily transmitted in aerosolized form [3],[4]. In addition, they have eluded systematic attempts at eradication for more than a century, even in most developed countries, and a human vaccine against brucellosis is not available [3]. Therefore, *Brucella* spp. have been classified as potential bioterror threat agents [5], and have generated significant interest in the biosecurity and world health communities.

Understanding the molecular mechanisms of *Brucella* pathogenesis and host response is critical for brucellosis control, and intracellular trafficking and replication of *Brucella* spp. play important roles in these processes [6]–[8]. First, bacteria, internalized from the host cell plasma membrane, orchestrate the biogenesis of early *Brucella-*containing vacuoles (BCVs) [9],[10]. Next, BCVs acidify but nevertheless fail to accumulate mannose 6-phosphate receptors (M6PRs) and cathepsin D, markers for late endosomes and lysosomes, respectively [8],[11]. Instead, maturing BCVs fuse with membranes that contain endoplasmic reticulum (ER) resident proteins, including calreticulin and calnexin [7],[8],[11]. In addition, this trafficking involves BCV interactions with a compartment that contains the autophagosomal marker monodansylcadaverin [7],[12]. Finally, *Brucella* spp. replicate in an ER-like compartment, and then presumably lyse the host cell to allow the infectious cycle to begin anew [8],[13],[14].

Bacterial lipopolysaccharides (LPS) play an important role in directing the bacterium along an intracellular trafficking pathway that enables a productive infection to be established. *Brucella* LPS also protects the bacterium from the harsh intracellular environment, suppresses pro-inflammatory and antibacterial host responses, and interferes with antigen presentation in macrophages [15]. Unlike their smooth wild-type (WT) counterparts, *B. melitensis* or *B. abortus* mutants harboring a deletion in the phosphomannomutase gene (Δ*manBA*) lack LPS O-antigens, form rough colonies on solid medium, and are rapidly internalized by macrophages via a poorly understood pathway [16],[17]. However, these mutants fail to establish an intracellular replicative niche and reportedly induce a necrotic cytopathic effect in these cells [18],[19]. The bacterial type IV secretion system (T4SS) is also important for bacterial pathogenesis, and mutant strains lacking this system fail to traffic to, or replicate in, the ER [7], [20]–[22].

To date, relatively few host factors, including Rho1, Rac1, Cdc42 [23] and Sar1 [8], have been shown to be important for *Brucella* infection. Phosphoinositide 3-kinase (PI3K) activities have also been implicated in supporting *Brucella* infection [23]. Despite these advances, factors that mediate *Brucella* infection of host cells remain obscure. However, *Brucella* intracellular trafficking from the plasma membrane to an ER-associated replicative niche involves interactions with a membrane bounded compartment that contains autophagosome markers [7],[12]. In addition, the organism replicates within a compartment that contains ER resident proteins [7],[8],[11]. These data thereby suggest that host cell autophagic pathway proteins, and ER-associated factors, may regulate the intracellular trafficking and replication of the pathogen.

Recent developments in the use of evolutionarily divergent *Drosophila* S2 cell model systems to study host-pathogen interactions, and RNA interference (RNAi) technology for knocking down host gene expression, have provided unprecedented opportunities for making significant progress in elucidating *Brucella* host factors. *Drosophila* S2 cells are macrophage-like cells that recapitulate conserved aspects of innate immunity [24] and that have been exploited for studying mammalian host-pathogen interactions. RNAi-based forward genetic screens in S2 cells have, for example, identified novel host factors involved in the recognition and replication of significant human bacterial pathogens, including *E. coli* [25], *Listeria* [26],[27], *Mycobacterium* [28], *Legionella* [29], and *Chlamydia* [30],[31]. Importantly, mammalian orthologs of hits identified in these screens have been shown to be important for bacterial infection of mammalian cells, thereby validating the utility of this *Drosophila* cell model for host-pathogen studies [28]–[31]. In this study, we show that the *Drosophila* S2 cell-*Brucella* interaction system recapitulates critical aspects of *Brucella* infection of mammalian cells. In addition, we demonstrate the power of this system by identifying novel *Brucella* host factors, including IRE1α, a conserved transmembrane kinase that plays a key role in regulating the host cell unfolded protein response (UPR) [32]–[34]. Finally, we demonstrate that IRE1α is required for *Brucella* infection of mammalian cells, and discuss a possible mechanism by which this intriguing protein may regulate bacterial infection.

## Results

### *Brucella* infection of *Drosophila* S2 and mammalian cells shares striking similarities

If *Drosophila* S2 cells are to provide a model system for studying *Brucella* infection, then they must support bacterial entry and replication. In addition, isogenic *Brucella* mutants with established entry, intracellular trafficking and replication properties should behave similarly in S2 cells and mammalian macrophages. Finally, *Brucella* should display similar infection phenotypes in S2 and mammalian cells that have been treated with compounds that disrupt host cell functions. With these ideas in mind, we employed gentamicin protection assays [18] to examine the entry and replication of different *B. melitensis* and *B. abortus* WT and mutant strains (listed in Table S1) in S2 cells. Because S2 cells require temperatures below 30°C for growth, all infection experiments were performed at 29°C, unless otherwise indicated. Importantly, J774A.1 cells supported *Brucella* entry and intracellular replication at this temperature (Fig. S2).

**Figure 1:**
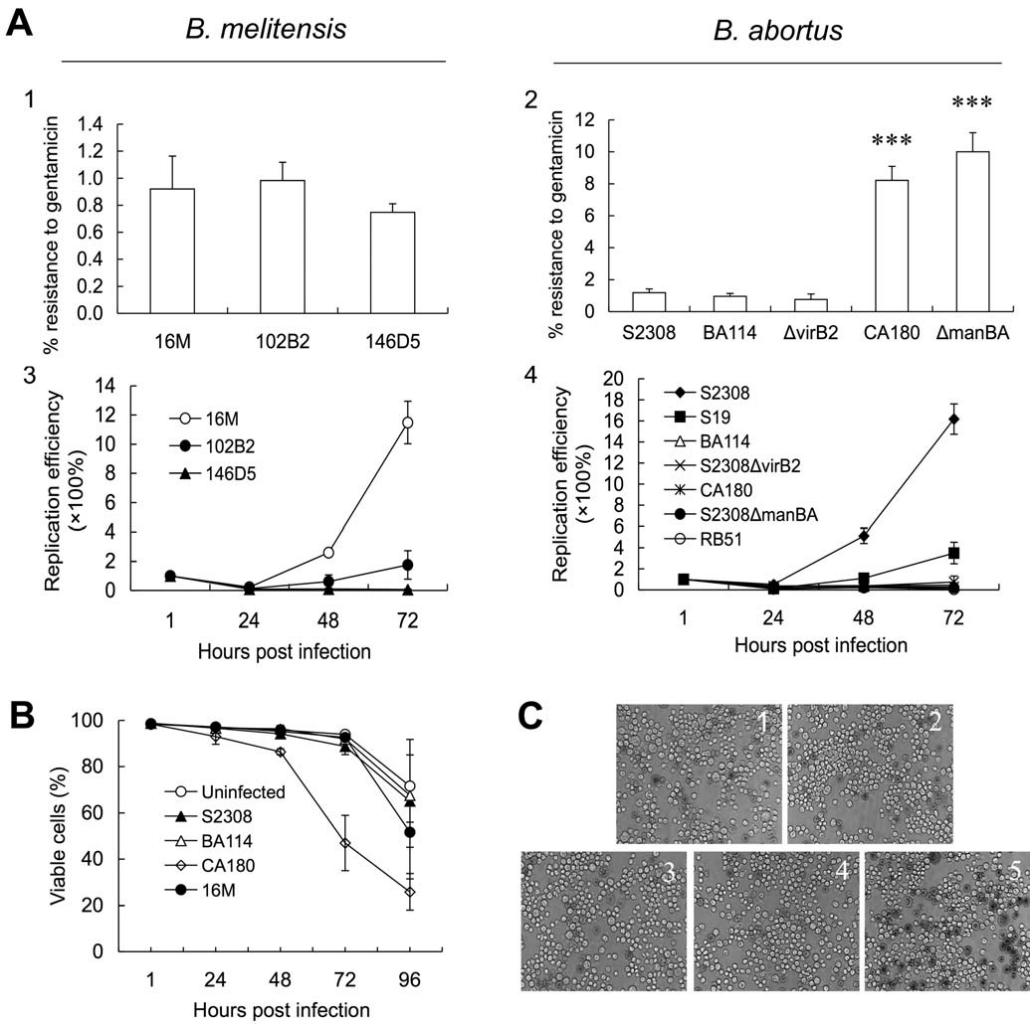
*Brucella abortus* and *B. melitensis* entry, replication and cytotoxicity in *Drosophila* S2 cells. A. Gentamicin protection assays were employed to assess the entry (panels 1 and 2) and replication (Panels 3 and 4) of assorted *B. melitensis* (Panels 1 and 3) and *B. abortus* (panels 2 and 4) strains. B. Rough mutant strains (CA180, S2308*manBA::Tn5*) induce a cytopathic effect in S2 cells. Trypan blue dye exclusion assays were used to measure the viability of S2 cells after infection with various *Brucella* strains. The *manBA* mutant induced a cytopathic effect on S2 cells. Smooth wild-type strains (16M and 2308) and strains lacking the T4SS VirB system (BA114, S2308*virB10::Tn5*) did not induce a similar effect. C. Viability of *Brucella* infected S2 cells at 48 h.p.i. *Drosophila* S2 cells were infected with *Brucella* strains, (1) uninfected control, (2) 16M, (3) S2308, (4) BA114, and (5) CA180 at an MOI of 200. Infected cells were stained with trypan blue, fixed and analyzed by Olympus IX70 inverted microscopy (magnification, ×200). Dead cells appear black. The images were taken from a representative experiment. All data represent the means ± standard deviations from at least three independent experiments.

*Brucella* WT (S2308 and 16M) and mutant strains displayed strikingly similar properties when infecting S2 and mammalian cells. First, *B. abortus* and *B. melitensis* strains with smooth colony morphologies (i.e., 102B2, 146D5, BA114, S2308Δ*virB2*) (Fig. 1 A1 and A2) and attenuated rough mutants (i.e., CA180, S2308Δ*manBA* and 16MΔ*manBA*) displayed corresponding entry phenotypes in *Drosophila* S2 and mammalian cells (Fig. 1 A2 and data not shown). Second, *B. melitensis* strains harboring mutations in *mucR* (strain 102B2) and *merR* (strain 146D5) failed to replicate in both J774A.1 [35] and S2 cells. *B. abortus* and *B. melitensis* strains lacking the T4SS (e.g., BA114, S2308Δ*virB2*, 16MΔ*virB2*) behaved similarly (Fig. 1 A3, A4 and data not shown). Third, vaccine strains RB51 and S19 [36] displayed significantly decreased levels of replication in both host cell systems (Fig. 1 A4 and data not shown). Fourth, similar cytopathic effects were observed when rough strain CA180 infected S2 and J774A.1 cells [18],[19] (Fig. 1B and 1C). Finally, the number of bacteria that entered S2 cells was directly proportional to the multiplicity of infection (MOI) (Fig. S3). This feature was also observed in mammalian cell systems (data not shown).

To easily visualize the intracellular trafficking and replication of *Brucella* spp., we exploited a GFP-expressing 16M strain (henceforth 16M-GFP) (Fig. S4). A comparison of the intracellular trafficking of *Brucella* spp. in S2 and mammalian cells indicated that the pathogen follows similar pathways in both host cell systems. BCVs trafficked to and replicated within an intracellular compartment that contained ER markers (e.g., mSpitz in S2 cells) [37], and was closely associated with COPII-coatomer (Sec 23) proteins (Fig. S5A and data not shown) in both cell systems. Quantitative analysis also demonstrated that the bacterium failed to accumulate late endosome, Golgi marker (dGRASP) [38], or lysosomal markers in S2 or mammalian cells ([7],[8],[12] and Fig. S5B). In addition, heat killed, formaldehyde fixed, and Δ*virB* controls did not similarly colocalize with ER markers in either system (Fig. S5A and data not shown). Therefore, the intracellular trafficking of *B. abortus and B. melitensis* in S2 and mammalian cells shared striking similarities.

Similar infection profiles were observed when *B. abortus* was used to infect mammalian or S2 cells that were treated with several compounds; these compounds disrupted host cell functions and did not impair the bacterial growth in culture, or the viability of infected S2 cells (Fig. S6). These included: cytochalasin D [23], a compound that disrupts actin polymerization; bafilomycin A1, a specific inhibitor of vacuolar H^+^-ATPase activity and endolysosomal acidification [39]; brefeldin A (BFA), a fungal metabolite that prevents the assembly of COPI coated vesicles and disrupts vesicular transport [7],[8] (Table S2 and Fig. S7A and S7B). Treatment of S2 and J774.A1 cells with the PI3K inhibitor wortmannin (WM) significantly reduced entry of *B. abortus and B. melitensis* (Fig. S7A and data not shown). However, WM treatment of S2 and J774.A1 cells had no effect on the replication efficiency of the internalized bacteria (Fig. S7C, and data not shown). These findings were similar to those previously reported in mammalian cell systems [8],[23],[39],[40]. In addition, we performed several experiments to assess the role of sphingolipids in supporting bacterial infection, and exploited myriocin (MR), a potent inhibitor of serine palmitoyltransferase (SPT), the first step in sphingosine biosynthesis [41], for these studies. *B. abortus* entry and survival were significantly inhibited when cells were treated with high MR concentrations (≥1 μM). Low concentrations (≤100 nM) of the compound had no effect on bacterial entry (Table S2 and Fig. S7A). However, the replication efficiency of the pathogen was decreased under these conditions (Fig. S7A).

### RNAi-mediated inactivation of host factors required for *Brucella* infection

**Figure 2:**
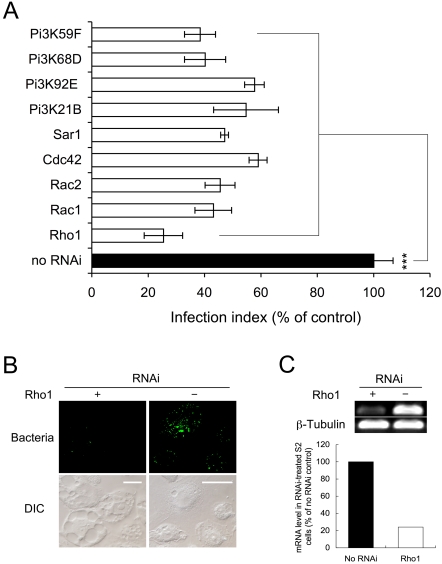
Evolutionarily conserved host factors mediate *Brucella* infection of *Drosophila* S2 cells. A. S2 cells that had been subjected to dsRNA-mediated gene depletion were infected with *Brucella* 16M-GFP at an MOI of 50. At 72 h.p.i., the cells were then replated onto ConA-coated 96 well glass bottom plates, washed three times with 1×PBS, fixed, stained and viewed with an Olympus IX70 inverted microscope. For each experiment, two images containing a total of at least 1,000 cells were analyzed using NIH Image J software and the infection index was determined. Data represent the means ± standard deviations from three independent experiments. *** indicates significance at P<0.001. B. dsRNA-mediated Rho1 knockdown in S2 cells results in large sized cells with reduced ability to support WT *Brucella* (S2308) infection (48 h.p.i., Scale bar: 10 μm). C. Reduction of Rho1 mRNA levels in dsRNA-treated S2 cells (upper panel) was assessed by RT-PCR and quantified by scanning densitometry (lower panel).

We employed RNAi technology to examine whether host proteins that are known to support bacterial infection of mammalian cells play similar roles in S2 cells. The evolutionarily conserved host proteins Rho, Rac, Cdc42 and Sar1 have been previously shown to be required for *Brucella* infection of mammalian cells [8],[23], therefore, we examined whether these proteins were also required for *Brucella* entry and replication in S2 cells. Fluorescence microscopy image assays were employed for these studies because they offered a rapid and convenient method for assessing bacterial infection. Importantly, similar results were obtained when either fluorescence microscopy or gentamicin protection assays were performed (Table 1 and Fig. 2A). When S2 cells were depleted of Rac and Cdc42, the entry of *B. abortus* (S2308) or *B. melitensis* (16M) was impaired (Table 1 and Fig. 2A). Rho1-depleted S2 cells appeared larger than untreated controls, contained numerous enlarged intracellular vacuoles, and also displayed significantly decreased levels of *Brucella* entry (Table 1, Fig. 2B). Sar1-depleted S2 cells also displayed dramatically reduced levels of *Brucella* replication (Table 1, Table S3 and Fig. 2A) were observed in these cells. These findings were similar to results obtained when *B. abortus* was used to infect mammalian cells in which the activities of the corresponding human orthologous proteins had been depleted [8],[23]. Therefore, the activities of these evolutionarily conserved GTP-binding proteins were required to support bacterial infection of both S2 and mammalian cells (Table 1, Table S3, Fig. 2 and Fig. S8).

**Table 1:** Evolutionarily conserved host factors required for *Brucella abortus* (S2308) infection in both *Drosophila* S2 and mammalian cell lines

Gene name	CFU (% of control) a						Reference
	S2 cells				Mammalian cells		
	CG #	Method	Internalization	Replication	Method	Internalization	
Rho1	8416	dsRNAi	25.1±15.2** b	22.4±11.2**	Dom-neg	∼28±5	[23], this study
Rac1	2248	dsRNAi	ND d	63.7±13.0** e	Dom-neg	∼37±3	[23], this study
Rac 2	8556	dsRNAi	45.9±11.1**	46.0±16.5**	ND	ND	This study
Cdc 42	1253	dsRNAi	51.9±24.6**	56.0±19.5**	Dom-neg	∼45±17	[23], this study
Sar 1	7073	dsRNAi	ND	47.1±1.3** e	Dom-neg	NA^f^	[8], this study
Pi3K21B	2699	dsRNAi	59.7±10.8**	65.9±7.5**	p85αβΔ g	9.7±2.7**	This study
					p85β^−/−^	43.7±5.5**	This study
Pi3K92E	4141	dsRNAi	62.2±14.9**	65.7±5.2**	ND	ND	This study
PI3Ks (2) h		dsRNAi	59.5±14.7**	60.4±15.8**	ND	ND	This study
Pi3K68D	1162	dsRNAi	ND	47.7±4.8**	ND	ND	This study
Pi3K59F	5373	dsRNAi	49.6±10.5**	44.4±16.9**	ND	ND	This study
PI3Ks (4) i		dsRNAi	64.4±9.0**	52.8±5.3**	ND	ND	This study

a Colony forming units (CFUs) of the untreated control was normalized as 100%.

b Data represent the means±standard deviations from at least three independent experiments.

**: represents significant at P<0.001 compared with no-RNAi control.

c Dominant-negative mutant.

d Not detected.

e Data resulted from image analysis using NIH Image J software describe in *Materials and Methods*.

f No accurate data, ∼30% of infected cells with replicating bacteria.

g MEFs are deficient in class IA PI3Ks p85α and p85β (p85α^−/−^p85β^−/−^).

h Double strand RNA targeting to the two PI3Ks Pi3K21B (CG2699) and Pi3K92E (CG4141) in class I_A_ and I_B_, respectively.

i Double strand RNA targeting to all the four members of PI3Ks [Pi3K21B, Pi3K92E, Pi3K68D (CG11621, class II PI3K) and Pi3K59F (CG5373, class III PI3K)] in the fly genome.

**Figure 3:**
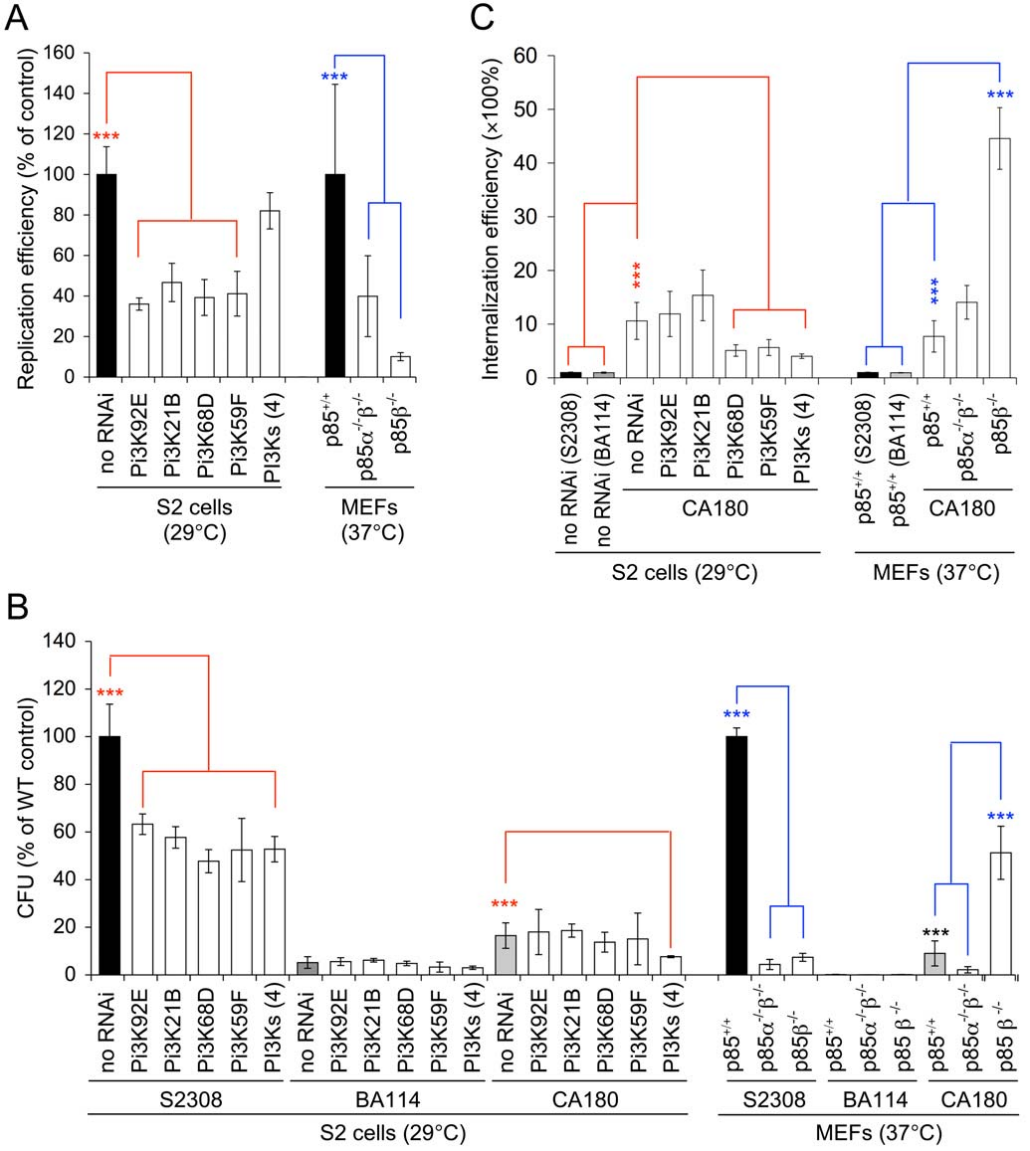
Depletion of individual host phosphoinositide 3-kinases (PI3Ks) affects *Brucella abortus* infection of *Drosophila* and mammalian host cells. A. Replication efficiency of WT *Brucella* (S2308) in depletion of PI3Ks of S2 cells (72 h.p.i.) and MEFs (48 h.p.i). B. Relative bacterial number (CFU) in *Brucella* infection of PI3K-depleted cells at 72 h.p.i. (S2 cells) or 48 h.p.i. (MEFs). C. Depletion of PI3Ks affects *Brucella* rough mutant CA180 entry into *Drosophila* and mammalian host cells. For these experiments, either *Drosophila* S2 cells, treated with dsRNAs to deplete individual PI3Ks, or mouse embryonic fibroblasts (MEFs) harboring deletions of the two regulatory isoforms of class I_A_ PI3Ks (P85α and P85β), were infected with *Brucella* rough mutant CA180. The infected cells were lysed and subjected to gentamicin protection assays after an additional 1 hr of incubation with media supplemented with 40 μg/ml gentamicin at the indicated temperatures. The CFUs of all the treatments at the indicated time points were compared with that of the WT (S2308) control. CFUs were counted after 3 days of incubation at 37°C. Data represent the means ± standard deviations from three independent experiments. *** indicates significance at P<0.001.

To assess whether PI3Ks played similar roles in supporting bacterial infection of mammalian and S2 cells, we performed several experiments. First, we treated S2 and J774A.1 cells with WM and found that the levels of *B. abortus* and *B. melitensis* entry decreased in a similar fashion in both host cell systems (Table S2, Fig. S7A and data not shown). Second, we employed RNAi technology to deplete S2 cells of individual PI3K proteins and then measured bacterial entry and replication. These experiments revealed that multiple classes of PI3Ks are required to support *B. abortus* and *B. melitensis* WT strain infection (Table 1, Table S3, Fig. 2A, Fig. 3A and 3B). However, rough and smooth strains exploit separate host molecular pathways for entry [42]. when *B. abortus* rough strain CA180 was used to infect PI3K-depleted S2 cells, bacterial entry was dramatically enhanced (Fig. 3C). These data indicated that multiple PI3Ks play differential roles in mediating the entry of smooth and rough *Brucella* strains into S2 cells.

### Experiments in mammalian cells confirm results obtained in *Drosophila* S2 cells

If *Drosophila* S2 cells are to serve as a useful model host cell system, then results obtained using this system should mirror corresponding mammalian cell findings. To test this possibility, we examined whether a murine ortholog (p85) of a model *Drosophila* gene (Pi3K21B) that supports *Brucella* infection of insect cells (Table 1, Table S3, Fig. 2A, Fig. 3A and 3B) mediates bacterial infection of murine cells. We used immortalized mouse embryonic fibroblasts (MEFs) derived from knockout mice harboring deletions in class I_A_ PI3Ks (p85α and p85β) [43] for these studies. As expected, the levels of *B. abortus* and *B. melitensis* WT strains entry into MEF cells harboring PI3K gene deletions were dramatically reduced (Table 1 and data not shown). p85α^−/−^ p85β^−/−^ and p85β^−/−^ MEFs supported lower levels of *B. abortus* and *B. melitensis* WT strains entry than p85^+/+^ controls (Table 1 and data not shown). However, when these MEFs were infected with *Brucella* rough mutants (CA180 and S2308Δ*manBA*), bacterial internalization significantly increased, especially in p85β^−/−^ MEFs (Fig. 3C and data not shown). These findings were similar to results obtained in experiments in which the entry of a *Brucella* rough mutant into class I_A_ PI3K-depleted S2 cells was examined (Fig. 3C). Therefore, host cell PI3K isoforms differentially mediated the infection of smooth and rough organisms in both cell systems, and supported the use of the *Drosophila* cell system for elucidating novel *Brucella* host cell factors.

### RNAi screen for ER-associated host factors

We were encouraged by our findings that previously described mammalian host proteins (i.e., Rho1, Rac, Cdc42 and Sar1) played similar roles in S2 cells. In addition, we noted that the *Drosophila* S2 cell system enabled the first molecular dissection of host cell PI3K isoform activity during *Brucella* infection. We therefore examined whether the *Drosophila* S2 cell system and RNAi technology could be combined to identify novel *Brucella* host factors. To focus our experiments, we constructed and screened 240 dsRNAs, including 110 dsRNAs that targeted the knockdown all of the genes annotated to be associated with the ER in the *Drosophila* RNAi Library Release 1.0 (Open Biosystems, Huntsville, AL, USA). The ER was ripe for examination because *Brucella* is known to replicate within a poorly characterized ER-like compartment, thereby suggesting that ER-associated host factors may be involved in regulating the intracellular replication of the pathogen.

**Figure 4:**
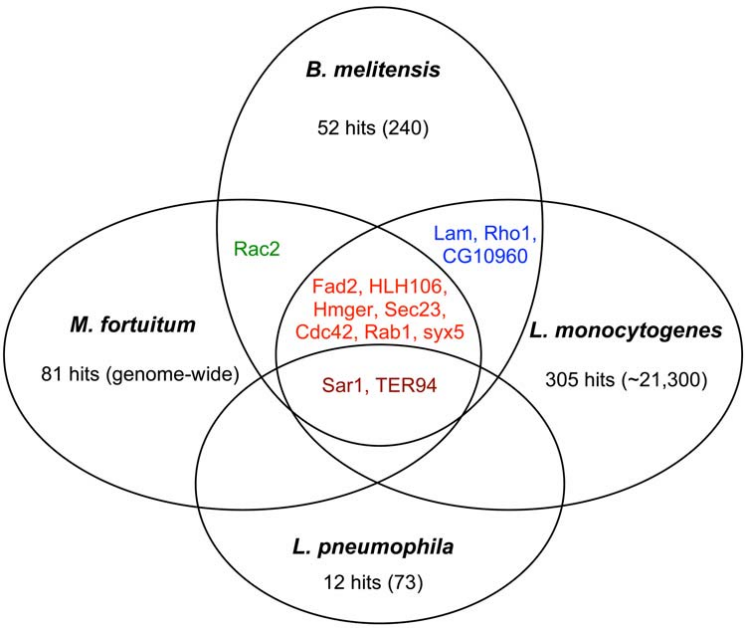
Host factors mediating intracellular bacterial infection. Shared and unique host factors were determined by analyzing published data from screens performed in S2 cells infected with *Mycobacterium fortuitum* [28], *Listeria monocytogenes* [26],[27], *Legionella pneumophila* [29] and *Chlamydia caviae* [30]. Numbers in parenthesis represent the number of screened or targeted genes.

Our ER-directed RNAi screen gave several interesting results. First, our screening approach successfully identified 52 hits. A hit was defined as a sample in which the relative infection differed by more than two standard deviations from the untreated control (Table S3). Importantly, control genes (i.e., Rho1, Rac, Cdc42, Sar1 and PI3Ks) were identified as hits in the screen (Table S3). Therefore, our screening strategy was sufficiently robust to uncover known or suspected host factors. We were curious whether the hit frequency obtained in our ER-targeted screen would be the same if a set of dsRNAs that were not associated with the ER were screened. We therefore screened 130 dsRNAs that were randomly picked from 2 of the 76 96-well plates in the *Drosophila* RNAi library. Because the manufacturer randomly arrayed dsRNAs into the source plates, this strategy for picking dsRNAs to be screen introduced no bias in the functions of the targeted genes in the screen. Notably, this experiment uncovered only 2 hits (∼1.5% of the total) (Table S3), and therefore gave a hit frequency that was comparable to that observed in the *Mycobacterium fortuitum* and *Listeria monocytogenes* whole genome RNAi screens [26]–[28]. Interestingly, 14 out of 52 hits in our screen had been previously shown to mediate infection of S2 cells by *Mycobacteria*, *Listeria*, *Legionella* and *Chlamydia* infection [26]–[31] (Table S3 and Fig. 4). On the other hand, 29 genes were identified that had not been previously reported to be involved in supporting intracellular bacterial infection (Table S3). These novel genes were classified according to the gene ontology system of biological and molecular function, cellular component, or protein domains as reported in FlyBase (www.flybase.org). This classification revealed that the novel hits represented a variety of functional classes, including kinases, chaperones, and biosynthetic/metabolic enzymes. In addition, these 29 genes were localized to either the ER lumen (CG9429, CG30498) or ER membrane (CG6437, CG1063) (Table S3). We re-tested some of our most interesting hits in both fluorescence microscopy and gentamicin protection assays (Table S3, repeat≥3 times), and also employed quantitative reverse transcriptase polymerase chain reaction (Q-PCR) to verify that the expression of these genes in S2 cells was knocked down by dsRNA treatment. We typically obtained 60–90% knockdown of target gene expression in our screening plates (Fig. 2C and data not shown).

**Figure 5:**
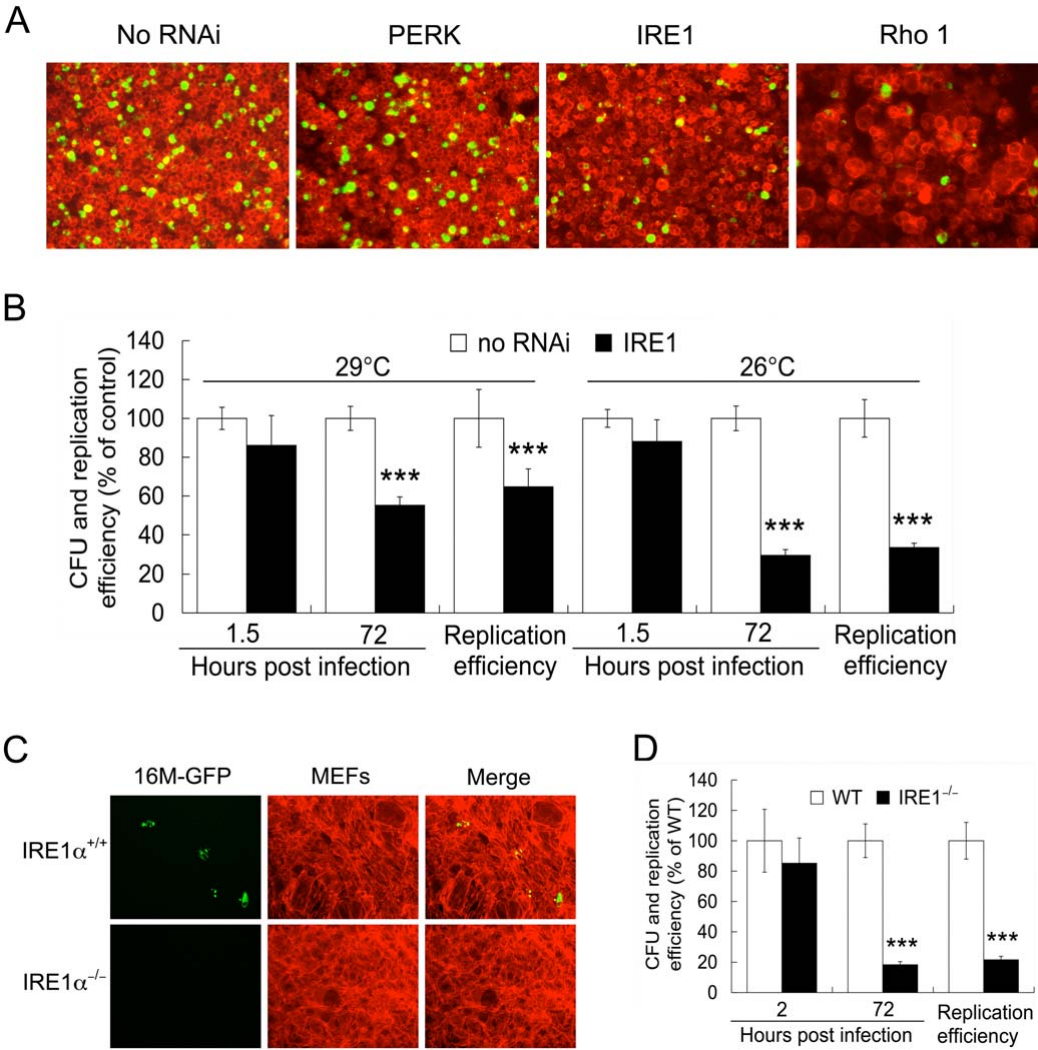
IRE1α is required for *Brucella melitensis* infection. A. *Brucella* (16M-GFP) infection of S2 cells in which the expression of the indicated host genes has been knocked down by dsRNA treatment at 72 h.p.i.. No RNAi-treated cells, and cells in which Rho1 expression was knocked down by dsRNA treatment were used as positive and negative controls, respectively. B. Depletion of IRE1 expression in S2 cells inhibits 16M-GFP replication. IRE1 has a limited role in *B. melitensis* entry S2 cell. C. *B. melitensis* 16M-GFP infection (MOI = 100) of mouse embryonic fibroblast (MEF) IRE1α-null and their wild-type counterparts at 48 h.p.i. D. IRE1α^−/−^ MEF cells support *B. melitensis* entry but not replication. *** Significant at P<0.001. Data in B and D represent the means ± standard deviations from three independent experiments. The images in A and C were taken from a representative experiment.

Although each screen hit constituted a potential entry point for investigating the mechanism by which *Brucella* secures a replicative niche, we were particularly intrigued with IRE1 (CG4583), a key signal transducer that plays an important role in regulating the host cell UPR [32]–[34]. RNAi mediated knockdown of IRE1 gene expression resulted in significant reductions in *Brucella* replication (Fig. 5A, 5B and Table S3). In addition, IRE1 had not been previously implicated as a bacterial host factor. These data raised the intriguing possibility that IRE1 may play a novel role in regulating *Brucella* infection. We therefore examined whether IRE1α (the mammalian ortholog of *Drosophila* IRE1) was important for *Brucella* infection of mammalian cells.

### IRE1α is required for efficient *Brucella* replication in mammalian cells

**Figure 6:**
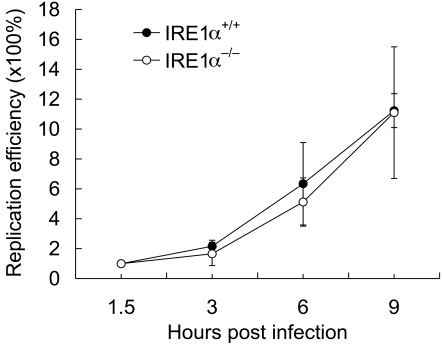
IRE1α is not required for *Salmonella* entry and replication. MEF IRE1α^+/+^ and IRE1α^−/−^ cells were infected with *Salmonella enterica* serovar *typhi* wild type strain SL1344 at an MOI of 50. Gentamicin protection assays were employed to assess *Salmonella* entry [At 1.5 h.p.i, the number of CFUs for IRE1α^+/+^ and IRE1α^−/−^ was (2.49±0.36)×10^6^ and (2.23±0.22)×10^6^, respectively] and replication (3, 6 and 9 h.p.i). All data represent the means ± standard deviations from three independent experiments.

We performed several experiments to examine whether IRE1α played a critical role in supporting *Brucella* infection of mammalian cells. First, we infected IRE1α-null (IRE1α^−/−^) and WT (IRE1α^+/+^) control MEF cells with 16M-GFP (Fig. 5C), and also performed gentamicin protection assays to assess bacterial entry and replication (Fig. 5D). The level of bacterial entry in IRE1α^−/−^ was not statistically different from IRE1α^+/+^ controls (Fig. 5D). However, bacterial replication was significantly inhibited in IRE1α-depleted S2 cells and in IRE1α-null MEF cells (Fig. 5). Trypan blue dye exclusion analysis of 16M-infected MEF cells failed to reveal differences in host cell survival (data not shown). Therefore, the differences in bacterial replication efficiencies in these cell lines were not caused by the induction of host cell pro-apoptotic programs or by differences in the survival of *Brucella*-infected IRE1α^−/−^ MEFs. Instead, they appeared to reflect a specific and important bacterial requirement for host cell IRE1α activity. Finally, the levels of entry and replication of *Salmonella enterica* serovar *typhi*, and the amounts of latex bead internalization, were similar in control and IRE1α^−/−^ cells (Fig. 6 and data not shown). These data supported the idea that IRE1α^−/−^ cells do not possess general defects in phagocytosis, and that IRE1α activity is not required to support infection by all intracellular bacterial pathogens (Table S3 and Fig. 6).

### PERK, ATF6 and BBF-2 are not required for *Brucella* replication

**Figure 7:**
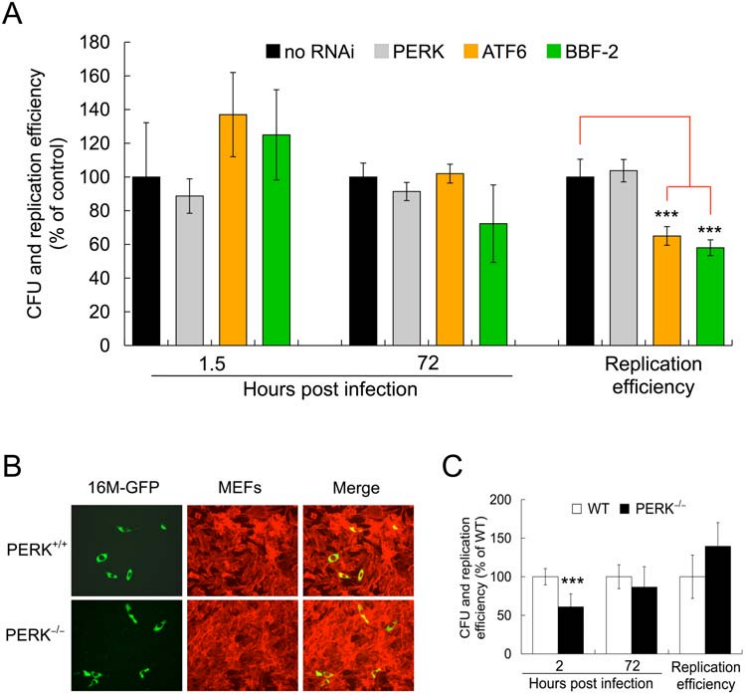
Endoplasmic reticulum UPR regulators PERK, ATF6 and BBF-2 display limited roles in *Brucella melitensis* infection. A. Depletion of PERK, ATF6 and BBF-2 in S2 cells displays no significant difference in the number of bacteria (16M) (CFU, % of control) in entry (1.5 h.p.i) and replication (72 h.p.i), although the replication efficiency of depletion of ATF6 and BBF-2 displayed significantly reduced. B. Infection of mouse embryonic fibroblast (MEF) PERK^−/−^ and their WT counterparts by *B. melitensis* 16M-GFP (MOI = 100) displays no significant difference at 48 h.p.i. C. PERK^−/−^ MEF cells have no effect on *B. melitensis* replication although the entry of the bacterium displays significant difference. *** Significant at P<0.001. Data in A and C represent the means ± standard deviations from three independent experiments. The images in B were taken from a representative experiment.

Besides IRE1α, several other ER-associated transmembrane signaling molecules play important roles in initiating and regulating UPR in host cells, including PERK, ATF6, and BBF2H7 (mammalian BBF-2 ortholog) [32]–[34], [44]–[46]. We performed fluorescence microscopy and gentamicin protection assays to examine their role in *Brucella* entry and replication. *B. melitensis* (16M) entry and replication in PERK-, ATF6-, and BBF-2-depleted S2 cells were not significantly different from untreated controls (Fig. 5A, Fig. 7A and Table S3). Although *B. melitensis* replication efficiency decreased in ATF- and BBF-2-depleted S2 cells, the number of bacterial colony forming units (CFUs) in these cells at 72 hours post-infection (h.p.i.) was not significantly smaller than controls (Fig. 7A and Table S3). Importantly, bacterial replication efficiencies in PERK^−/−^ and PERK^+/+^ MEF cell lines were similar (Fig. 7B and 7C). Taken together, these results demonstrated that not all UPR signaling molecules are required to support the replication of this pathogen, and that IRE1α plays a specific role in this process.

## Discussion

The study of host-*Brucella* interactions has suffered from the absence of a tractable genetic system to elucidate host factors. However, data obtained in this study indicate that *Drosophila* S2 cells provide a compelling model system for identifying and characterizing these important proteins. *Brucella* infection of *Drosophila* S2 cells recapitulates important aspects of mammalian cell infection. First, isogenic mutants of *Brucella* spp. behaved similarly in S2 and mammalian cells. In addition, these divergent host cell systems displayed similar trends in infection by smooth and rough strains with varied pathogenicity. *Brucella* rough mutants, such as CA180, were cytopathic to both mammalian and S2 host cells [18],[19, this study]. Therefore, these cells share conserved molecular mechanisms for recognizing and responding to *Brucella* LPS mutants. Second, *Brucella* entry and replication in S2 and mammalian cells were similarly sensitive to pharmacological perturbation by structurally diverse compounds. Of particular interest was the observation that MR, an inhibitor of STP, the rate-limiting enzyme in sphingolipid biosynthesis, dramatically reduced the amount of *Brucella* infection of S2 cells (this study). Previous studies have demonstrated an important role for sphingolipid enriched lipid rafts in pathogen infection [47]–[50], and our MR experiments support these observations. Third, *Brucella* infection of S2 cells required the activities of conserved GTP-binding proteins (Rho1, Rac, Cdc42, and Sar1), suggesting that *Brucella* infection of mammalian and *Drosophila* cells shared similar host molecular requirements. Finally, the activities of PI3Ks differentially regulate smooth and rough *Brucella* infection in both mammalian and *Drosophila* S2 cells (this study). Interestingly, the effects of PI3K knockdown in MEF cells were more dramatic than in S2 cells. In MEFs, PI3K genes are deleted, and thus the corresponding enzyme activities are absent. However, in *Drosophila* S2 cells, PI3K gene expression is knocked down (60–90%), and some residual activity may remain. These differences likely account for the differential infection of these cell types. Taken together, our data support the conclusion that S2 cells provide a useful model for investigating host-*Brucella* interactions.

Our demonstration that *Drosophila* S2 cells can be used to illuminate *Brucella* host factors is surprising because *Brucella* spp. do not occupy a described environmental niche outside of the mammalian host. In addition, the bacteria do not grow well in culture at temperatures below 35–37°C. However, previous reports have demonstrated *B. suis* multiplication within U937 cells at 30°C [51]. Therefore, *Brucella* growth below 37°C is not restricted to *B. melitensis* and *B. abortus* strains. Second, *Brucella* replication in J774A.1 and *Drosophila* S2 cells at 29°C share similar kinetics (Fig. S2B and S2C). Although a difference in the replication efficiency of S2308Δ*virB2* in J774 and S2 cells at 24 and 48 h.p.i was observed, no difference was detected at 72 h.p.i. Therefore, the differential growth of *B. abortus* and *B. melitensis* in these host cell systems likely results from differences in the growth temperature, and not from differential subversion of conserved host cell functions. Third, the most important criterion for judging the utility of a model non-mammalian host-pathogen interaction system is whether it can be exploited to shed new insights into the interaction in mammalian cells. In this regard, it should be noted that bacterial pathogens, such as *Listeria monocytogenes* [52], grow more slowly in *Drosophila* S2 cells than in mammalian cells; however, many host factors required for entry and survival of these intracellular pathogens have been identified using *Drosophila* S2 cells as a platform [25]–[31]. We expect to garner similar insights through the use of our *Drosophila* S2 cell-*Brucella* interaction system, and our demonstration that PI3Ks and IRE1α mediate *Brucella* infection of *Drosophila* S2 cells and murine embryonic fibroblasts support this view.

Our RNAi screen in S2 cells for ER-associated *Brucella* host factors provides new insights into how *Brucella* secures an intracellular replicative niche. Our screen identified 52 genes that participate in this process, 29 of which had not been previously suggested to support bacterial pathogen infection. In addition, we dissected the role of 4 PI3K isoforms. The number of identified hits (50 out of 110 pre-selected ER- associated genes) was striking, and likely reflects that sustained and multi-faceted *Brucella*-ER interactions are required for *Brucella* replication in host cells. Interestingly, 14 of the genes identified in our screen were also required for infection of S2 cells by other intracellular bacterial pathogens, including *Listeria*, *Mycobacteria*, *Legionella* or *Chlamydia* [26]–[31]. The fact that *Brucella* and *Legionella* share several ER-associated host factors is perhaps not surprising, especially given that both organisms engage in sustained interactions with the host ER as part of their virulence and replication programs [53],[54]. Finally, *Brucella*-specific ER-associated factors, such as IRE1 (CG4583), were uncovered in our screen. IRE1 may constitute a species-specific host factor that plays a role in mediating the unfolded protein response, thereby suggesting that the modulation of this stress-response system may be critical to bacterial intracellular survival and replication.

In eukaryotic cells, IRE1α mediated UPR induction is associated with enhanced expression of genes encoding ER chaperones and protein-folding catalysts, and proteins that participate in ER-associated degradation (ERAD) [55],[56]. IRE1α activation also induces the biosynthesis of membrane phospholipids that increase the surface area and volume of rough ER [57],[58]. In *Brucella* infected cells, IRE1α mediated activity may result in the biosynthesis of ER membrane that can be exploited by the pathogen to expand the size and enhance the quality of its replicative niche However, our data indicate that other UPR signal transducers, including PERK, are not required for *Brucella* infection in both *Drosophila* S2 and murine embryonic fibroblast cell systems. Therefore, not all UPR regulatory proteins are important for bacterial replication (Fig. 7 and Table S3), raising questions about the privileged status of IRE1α among these classes of molecules.

Recent reports have indicated an intriguing link between IRE1α activity and autophagic vacuole biogenesis [59],[60]. For example, IRE1α is required for the autophagy observed after cells are treated with the ER stress-inducing agents DTT, tunicamycin or thapsigargin [59],[60]. However, parallel experiments using PERK-deficient cells, and cells in which the expression of ATF6 had been knocked down, demonstrated that these UPR-associated signal transducers are not directly involved in the response to these drug treatments [60]. Therefore, IRE1α can regulate some autophagic events independently from input by these other ER associated signaling molecules.

**Figure 8:**
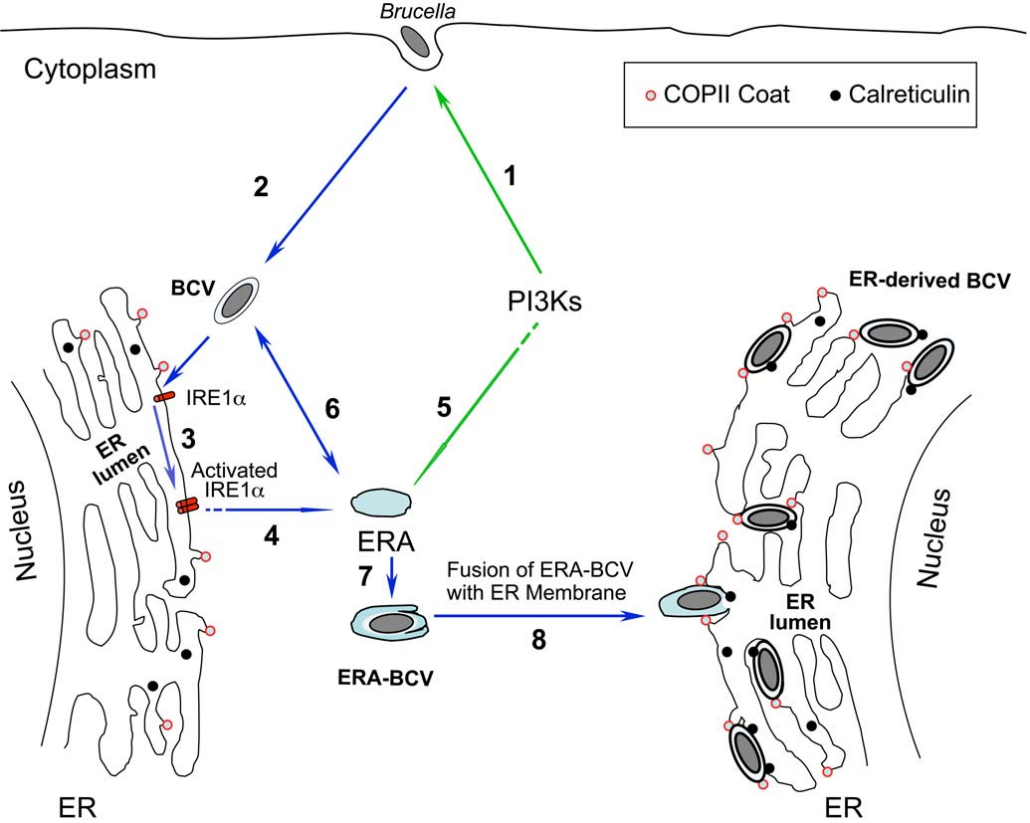
Model describing how *Brucella* may exploit IRE1α to secure a replicative niche in an ER-like compartment. After PI3K-dependent *Brucella* internalization (1), intracellular *Brucella*-containing vacuoles (BCVs) traffic to the ER in a T4SS-dependent fashion (2). Accumulation of BCVs in the ER may modulate IRE1α activities (3), which then may trigger the biogenesis of ER-containing autophagosomes (ERAs) [59]–[60] (4). ERA biogenesis is known to require the activities of early secretory pathway components, including members of the COPII complex (Sar1-Sec23-Sec24) [61]–[63] (indicated in red). This process is also regulated by the activities of PI3Ks [64]–[66] (5). ERAs may then fuse with BCVs (6) to form ERA-BCVs (7). In addition, ER expansion may occur in response to these events. Finally, ERA-BCVs may fuse with an expanded ER membrane (8) and intercept ER proteins such as calreticulin (indicated in black) to form ER-derived BCVs that are permissive for *Brucella* replication. Depletion of members of COPII complex, PI3Ks and ER proteins such as calreticulin disrupted these processes and dramatically reduced *Brucella* replication.

The differential participation of IRE1α, ATF6 and PERK in regulating the autophagy observed after cells are treated with stress-inducing agents is strikingly similar to their differential roles in mediating *Brucella* replication. IRE1α is required for *Brucella* to replicate efficiently; however, *Brucella* replication in PERK-, ATF6-, and BBF-2-depleted S2 cells was not significantly different from untreated controls. This differential participation therefore suggests a model in which IRE1α regulates *Brucella* infection by modulating the host cell autophagy pathway (Fig. 8).

Based on findings from our dsRNA screen, we propose a multi-step model by which IRE1α regulates *Brucella* replication. First, BCVs traffic to a compartment that contains ER resident proteins. Concomitantly, BCVs trigger IRE1α activation, which in turn, stimulates the biogenesis of ER-associated autophagosomes (ERAs) [59],[60]. ERAs then fuse with BCVs to form ERA-BCVs. This process is also regulated by the activities of PI3Ks. Finally, ERA-BCVs fail to fuse with lysosomes and hence avoid degradation; instead, they fuse with the ER to form ER-derived BCVs that are permissive for *Brucella* replication (Fig. 8).

Several pieces of evidence support this view. First, IRE1α, but neither PERK nor ATF6, is required for the induction of autophagy in response to treatment by ER stress-inducing agents [60]. Similar requirements for host proteins are observed during *Brucella* replication (Fig. 5, Fig. 7 and Table S3). Second, the assembly of ERAs is dependent upon early secretory pathway molecules [61]–[63]. In yeast, the COPII mutants *sec16*, *sec23*, and *sec24*, are defective in autophagy. However, mutations in two other COPII genes, *sec13* and *sec31*, do not affect ERA biogenesis and autophagy [61],[62]. In addition, PI3K activity is important for this process [64]–[66]. Our data demonstrate similar host factor requirements during *Brucella* infection of *Drosophila* cells. Specifically, depletion of Sec23, Sec24 and PI3Ks in host cells dramatically reduces *Brucella* replication (Table 1, Table S3, Fig. 2 and Fig. 3). However, depletion of Sec31 has no affect on this process (Table S3). Finally, *Brucella* trafficking to its intracellular replicative niche involves interactions with a compartment that contains the autophagosomal marker monodansylcadaverin [7],[12]. These localization data thereby establish a physical interaction between internalized *Brucella* and the host cell autophagy pathway. It should be noted, however, that although we cannot rule out the possibility that *Brucella* trafficking in MEFs differs from professional phagocytes, *Brucella* trafficking in HeLa cells and phagocytes share striking similarities [12]. Therefore, our observations in MEFs likely shed light on *Brucella* infection of phagocytes. Taken together, the data are consistent with the idea that IRE1α activity plays an important role in supporting *Brucella* interactions with the host cell ERA biogenesis machinery in mammalian cells (Fig. 8). Future studies will exploit the genetic power of the *Drosophila* S2 cell system to elucidate this intriguing possibility, and to define the precise molecular mechanisms by which *Brucella* secures an intracellular replicative niche.

## Materials and Methods

### Bacterial Strains

*Brucella melitensis* strain 16M (WT) and *B. abortus* strain 2308 (WT), and their derived mutants are listed in Table S1. Bacteria were grown in tryptic soy broth (TSB) or on tryptic soy agar (TSA, Difco™) plates, supplemented with either kanamycin (Km, 50 μg/ml), or chloramphenicol (Cm, 25 μg/ml) when required. For infection, 4 ml of TSB was inoculated with a loop of bacteria taken from a single colony grown on a freshly streaked TSA plate. Cultures were then grown with shaking at 37°C overnight, or until OD_600_≈3.0.

### Cell Culture

Murine macrophage J774.A1 cells, MEFs and HeLa cells were routinely cultured at 37°C in a 5% CO_2_ atmosphere in Dulbecco's Modified Eagle's Medium (DMEM) supplemented with 10% fetal bovine serum (FBS). S2 cells were maintained at 25°C in *Drosophila*-SFM medium or in Schneider's *Drosophila* medium (Invitrogen) supplemented with 10% FBS. Cells were seeded in 24-well plates and cultured overnight before infection. For antibiotic protection assays, 2.5×10^5^ cells were seeded in each well; for fluorescence microscopy assays (see below), 5×10^4^ cells were seeded on 12-mm glass coverslips (Fisherbrand) placed on the bottom of 24-well microtiter plates before infection.

### *Brucella* Infection

Host cells were infected with *Brucella* at an MOI of 100, unless otherwise indicated. Infected cells were then incubated at 29°C (S2 cells) or 37°C (mammalian cells) after centrifugation for 5 min at 200×g. Thirty minutes post-infection, culture media was removed, and the cells were rinsed with 1×phosphate buffered saline (PBS). Fresh media, supplemented with 40 μg/ml gentamicin, was then added for 1 hr to kill extracellular bacteria. Infected cells were continuously incubated in this antibiotic for various lengths of time at the indicated temperature. As indicated, viable bacteria in infected cells were analyzed using the antibiotic protection assay or the immunofluorence microscopy assay described below. In addition, *Brucella* replication efficiency ([# of CFUs at different time points post infection]/[# of CFUs of *Brucella* entry]) in the infected cells was also determined

### Antibiotic Protection Assays

At various times post-infection, viable bacteria present in infected cells were analyzed using gentamicin protection assays [18]. Briefly, infected cells were washed twice with 1×PBS buffer, lysed with 0.5% Tween 20 in sterile water, and the released bacteria were subjected to serial dilution in peptone saline [1% (wt/vol) Bacto peptone and 0.85% (wt/vol) NaCl]. Next, 10 μl of serial diluted cell lysate was plated on TSA plates. Finally, CFU were counted after three days of incubation at 37°C.

### Viability Assay of Infected Host Cells

S2 cells were coincubated with or without various drugs before 1 hr of and during *Brucella* infection (See below). Next, the infected cells were centrifuged at 200×g for 5 min and then incubated (30 min) with assorted *Brucella* strains. Fresh *Drosophila*-SFM media, supplemented with drugs (as indicated) and 80 μg/ml gentamicin, was added to kill extracellular bacteria. The infected and gentamicin treated cells were then incubated at 29°C for various lengths of time. To quantify the viability of S2 cells, at various time points, a portion of the infected cells was removed and processed for 0.2% trypan blue vital stain analysis. At least 500 cells were counted per sample. For image analysis, infected cells were replated onto ConA (Sigma)-coated 12-mm coverslips in 24-well plates and allowed to adhere for 1 hr. Cells were stained with 0.2% trypan blue for 5 min and then fixed with 1×PBS containing 3.7% formaldehyde for 1 hr. Viability of infected cells was assessed by analyzing images obtained with an Olympus IX70 fluorescence microscope. At least 500 infected cells per sample were used for the analysis.

### S2 Cell Transfection

To visualize *Brucella* spp. trafficking, S2 cells were transfected with ER marker mSpitz-GFP [37], and Golgi marker dGRASP-GFP [38] before infection. Specifically, S2 cells were grown to ∼80% confluence and then transfected using Effectene Transfection Reagent (Qiagen) as per the manufacturer's instructions. 0.25 μg of each pUAS-mSpitz GFP and pAcpA-Gal4 were employed in these transfection experiments. For the Golgi visualization experiments, 0.25 μg of dGRASP-GFP was used in the transfection. Typically, 1.5×10^6^ cells were transfected and then grown in 2.2 ml of Schneider's *Drosophila* medium supplemented with 10% FBS. Three days post-transfection, cells were replated onto ConA-treated 12-mm glass coverslips placed on the bottom of 24-well microtiter plates (for early time points of less than 8 hr) and immunofluorescence microscopy analysis was performed as previously described [18]. For later times points (≥8 hr), the transfected cells were reseeded directly in 24-well plates and allowed to adhere for 2 additional hours before infection with *Brucella*. At different post-infection time points, the infected cells were replated onto ConA-coated 12-mm coverslips and allowed to adhere for 1 hr. The cells were then washed three times with 1×PBS, fixed with 3.7% formaldehyde (pH 7.4) at room temperature for 1 hr and processed for immunofluorescence microscopy.

### Immunofluorescence Microscopy Assay

To elucidate *Brucella* spp. intracellular trafficking, S2 cells were infected with the following strains: *B. melitensis* (strains 16M or 16M-GFP); *B. abortus* (strain S2308); S2308 *virB2* deletion mutants; heat killed or 3.7% formaldehyde fixed WT strains. At various post-infection time points, S2 cells were replated onto ConA-coated 12-mm coverslips and allowed to adhere for 45 min to 1 hr. Cells were then washed, fixed as described above, and processed for immunofluorescence microscopy [18]. The primary antibodies used were as follows: goat polyclonal anti-*Brucella*; rabbit anti-human M6PR; rabbit anti-human cathepsin D; goat-anti rabbit Sec23 (COPII marker, Affinity BioReagents, Inc., CO, USA). Samples were stained with Alexa Fluor 488-conjugated and/or Alexa Fluor 594-conjugated donkey anti-goat/rabbit (Molecular Probes, 1:1000). Cover slips were then mounted in Vectashield mounting media (Vector Laboratories, Inc., CA, USA) and visualized with an Olympus BX51 confocal microscope. For quantitative analysis, single confocal section of random fields was acquired, and colocalization of markers was scored as positive when nonsaturated signals partially overlapped. Images for all immunofluorescence assays for *Brucella* spp. trafficking were acquired with a Hamamatsu ORCA-ER camera mounted on the Olympus BX51 microscope and driven by Simple PCI software (Compix Imaging Systems Inc., Cranberry Township, PA.). Images were processed with Adobe Photoshop CS Software (Adobe Systems Incorporated, San Jose, CA).

### Drug Treatments

*Drosophila* S2 cells or J774.A1 murine macrophages were coincubated in 24 well plates with assorted drugs including bafilomycin A1 (BAF), brefeldin A (BFA), cytochalasin D (CD), myriocin (MR) and wortmannin (WM) at the indicated concentrations. Cells were treated with drugs 1 hr before, and during, infection with the indicated *Brucella* strains. After infection, the treated cells were incubated at 29°C (S2 cells) or at 37°C with 5% CO2 (J774.A1 macrophages). To evaluate *Brucella* internalization, after 30 min of infection, fresh media, supplemented with the same concentration of the drugs and 80 μg/ml gentamicin was added to kill extracellular bacteria. After 45 min of incubation, the cells were lysed and the CFU per well determined by plating dilutions on TSA plates as described above. To assess *Brucella* intracellular replication, CFU analysis was performed at 72 h.p.i. The effect that BAF-mediated inhibition of host cell endosomal acidification exerted on *Brucella* replication was also examined. Briefly, BAF was added to the culture media 2 h.p.i. and continuously coincubated with infected cells for 72 hr. Cells were lysed and analyzed using the gentamicin protection assay. To investigate whether the drugs inhibit *Brucella* growth, the drugs were individually added to *Brucella* TSB cultures at 29°C or 37°C and incubated for 1 and 72 hr. CFU plating was used to assess bacterial growth in the presence of drugs, and thereby to evaluate the potential inhibitory effects.

### Generation of dsRNAs

Primers for generating RNAi that target the knockdown of *Drosophila* Rac1, Rac2, Rho1, Cdc42, Sar1 and PI3Ks were designed using sequence information present in flybase (http://flybase.org/). The primers were used in RT-PCR reactions to generate cDNAs. dsRNAs targeting genes to be knocked down were generated using previously described methods [26]. Briefly, gene-specific RNAi primers were used to amplify target sequences from *Drosophila* cDNA mixtures. The PCR products were re-amplified using the RNAi primers with T7 RNA polymerase promoter sequences in the 5′ end. The reamplified PCR products were then used as templates for the generation of dsRNAs. For generation of dsRNAs targeting ER-associated and other genes, cDNAs from commercially available *Drosophila* RNAi Library Release 1.0-DNA templates (Open Biosystems, Huntsville, AL, USA) were directly used as templates. One or two microliters (total ∼150 ng) of the PCR products were used to perform *in vitro* transcription reactions with the T7 MEGAscript kit (Ambion, Austin, TX) as per the manufacturer's instructions. Aliquots of *in vitro* transcription products were subjected to quality control by 1% agarose gel electrophoresis analysis and dsRNA concentrations were quantified using a NanoDrop® ND-1000 UV-Vis spectrophotometer (NanoDrop Technologies, Inc. Wilmington, DE).

### RNAi-mediated Gene Knock Down and Assays

1.0×10^6^ S2 cells were seeded in 12-well plates. dsRNAs (i.e., Rho1, Rac, Cdc42, Sar1 and PI3Ks) were added to each well at a final concentration of 15 μg/ml. After 4 days of incubation with dsRNA, an aliquot of the S2 cells was removed to check the efficiency of dsRNA mediated gene knock down by quantitative RT-PCR (Q-PCR). dsRNA-treated S2 cells in the same well were also re-plated in 24-well plates and allowed to adhere for at least 2 hr before infection. At the selected time points, the dsRNA-treated and *Brucella* infected cells were lysed and antibiotic protection assays or fluorescence microscopy image assays were performed as described.

To evaluate the utility of the combination of S2 cells and dsRNA technology, and the consistency of the results from antibiotic protection assays, we analyzed *Brucella* infection using fluorescence microscopy image assays. dsRNAs that target ER-associated genes or other known or unknown genes were added to 96-well microplates at a final concentration of 15 μg/ml (dsRNAs were added in duplicate in two different plates). S2 cells were then seeded in the plates with 5.0×10^4^ cells/well in 200 μl *Drosophila-*SFM medium. dsRNA-treated cells were incubated at 25°C for 4 days to allow for knockdown of target gene expression. The dsRNA-treated cells (100 μl) were replated into 96 well plates, infected with *B. melitensis* 16M-GFP at an MOI of 50. After 30 min of infection, the same amount of fresh media supplemented with 80 μg/ml gentamicin was added to each well and the infected cells were incubated at 29°C. At 72 h.p.i., infected cells were replated onto 96 well glass bottom plates (Greiner), that had been coated with ConA, and allowed to adhere for 1 hr. The infected S2 cells were washed 3 times with 1×PBS, fixed with 3.7% formaldehyde in 1×PBS at 4°C overnight, and stained with phalloidin-Texas red (1:1000) for 1 hr to visualize the host cell actin cytoskeleton. *Brucella* infected S2 cells were viewed with an Olympus IX70 inverted microscope and two 400× images from each well were acquired for image analysis. Images were analyzed using NIH Image J software (http://rsb.info.nih.gov/ij/), and the relative infection (RIF) [100×(% of infected dsRNA-treated cells)/(% of infected cells in the untreated control)] was determined. More than 1,000 S2 cells were counted to obtain the percentage of infection or infection index [(number of infected cells (at least 10 brucellae within the cell))/(number of total cells)] in a sample. The detailed process by which image analysis was performed is shown in Fig. S1. dsRNA screen was repeated once, and some of hits identified in both two round of screens were picked out to re-test in triplicate in fluorescence microcopy and gentamicin protection assay as described above.

### Mammalian Cell Infection

MEFs deficient of the two regulatory isoforms of class I_A_ PI3Ks (p85α^−/−^ p85β^−/−^ and p85β^−/−^) [43], IRE1α (IRE1α^−/−^) [67] and PERK (PERK^−/−^) [68] and their corresponding WT control p85^+/+^, IRE1α^+/+^ and PERK^+/+^ MEFs, were seeded in 24-well plates. After overnight culture, cells were infected with 16M-GFP and/or S2308, and their derived mutant strains. Infected cells were centrifuged for 5 min at 200×g and then incubated at 37°C for 60 min. Cells were washed with 1×PBS buffer, and fresh media supplemented with 40 μg/ml gentamicin was added. Cells were incubated for an additional 1 hr (entry) and 48 hr (replication) at 37°C. The amount of viable bacteria present in infected cells was assessed using gentamicin protection assays. For fluorescence microscopy and viability assays, 5×10^4^ cells were seeded onto 12-mm coverslips in 24-well plates. At 48 h.p.i., infected cells were subjected to the appropriate assays as described above.

### Statistical Analysis

All quantitative data were derived from results obtained in triplicate wells for at least three independent experiments. The significance of the data was assessed using Student's t-test, and all the analyzed data were normalized with internal controls before analysis.

## Supporting Information

Table S1 *Brucella* strains used in this study
(0.14 MB DOC)

Table S2 Comparison of *Drosophila* S2 and mammalian cells treated with drugs that inhibit *Brucella abortus* (S2308) entry and replication
(0.07 MB DOC)

Table S3 Effect of ER-associated cellular factors on *Brucella melitensis* replication
(0.10 MB XLS)

Figure S1 Schematic representation of image analysis using Image J to calculate the relative infection (RIF). 1. Threshold of bacterial replication in infected cells in an image using the same setting. 2. Analysis of particles in a thresholed image (i.e., the number of infected cells with bacterial replication). 3. Histogram of the image (cell numbers were adjusted via color density). 4. Calculation of the infection index and RIF (% of control) of the samples. For example, infection index of sample B31C07 = 39/68.69; RIF of B31C07 = 100×[39/68.69]/[106/68.41] = 36.12.
(8.15 MB TIF)

Figure S2 *Brucella abortus* and *B. melitensis* growth, and infection of host cells, at 29°C and 37°C. A. *B. abortus* growth in liquid culture (TSB) at 29°C and 37°C. B. Replication of *Brucella* wild-type strains S2308 and 16M in *Drosophila* S2 and J774.A1 murine macrophages at 29°C. The number of CFUs of 16M and S2308 for S2 [(4.18±0.54) ×10^5^/well and (3.51±0.95) ×10^5^/well, respectively] and for J774.A1 [(5.17±0.25) ×10^5^/well and (7.77±0.47) ×10^5^/well, respectively] cells at 1 h.p.i was normalized as 100%. *Brucella* replication efficiency was defined as the number of CFUs at different time points post infection/the number of CFUs of bacterial entry (1 h.p.i). C. Entry and replication of S2308 derived mutants in S2 and J774.A1 murine macrophages at 29°C. Data represent the means ± standard deviations from three independent experiments.
(0.50 MB PDF)

Figure S3 Infection of *Drosophila* S2 cells increases with rising multiplicity of infection (MOI). A. With increasing MOI, the number of S2 cells containing replicating *Brucella melitensis* (16M-GFP) increases at 72 h.p.i. The images were taken from a representative experiment. B. Infection index (i.e., Number of infected S2 cells with replicating *Brucella*/total cell number based on image analysis) and MOI display a linear relationship in the range of tested MOI. Data represent the means ± standard deviations from three independent experiments.
(10.28 MB TIF)

Figure S4 GFP expression has no effect on bacterial entry and replication. The entry (1.5 h.p.i) and replication (72 h.p.i.) of *Brucella melitensis* strains 16M and 16M-GFP in *Drosophila* S2 and J774.A1 murine macrophages at 29°C and 37°C, respectively, were compared using gentamicin protection assays. The number of 16M CFUs of entry and replication in *Drosophila* S2 cells [(4.85±0.46) ×10^5^/well and (8.89±1.23) ×10^6^/well, respectively] and in J774.A1 cells [(3.68±0.29) ×10^5^/well and (1.77±0.21) ×10^7^/well, respectively] were normalized as 100%. No significant differences in entry or replication in S2 or J774.A1 cells were observed in the two strains. Data represent the means ± standard deviations from three independent experiments.
(0.30 MB TIF)

Figure S5 *Brucella melitensis* intracellular trafficking in *Drosophila* S2 cells. A. 1. S2 cells expressing a GFP-tagged variant of the *Drosophila* ER maker mSpitz (mSpitz-GFP). 2. *B. melitensis* (16M) infection of S2 cells (24 h.p.i). 3. A tight association between *B. melitensis* cells and host cell ER membranes (arrow) in the main and inset panels is observed in the merged image. 4. Immunofluorescence localization of COPII in S2 cells using Sec23 polyclonal antibodies. 5. 16M-GFP localization in S2 cells at 12 h.p.i. (Green, Panel A). 6. A merged image showing COPII proteins and *B. melitensis* (16M-GFP) localization. 7. mSpitz-GFP localization in S2 cells. 8. Fixed and killed *B. melitensis* (16M) in S2 cells at 24 h.p.i. 9. Merged panels 7 and 8. Markers used in the panels indicated in parenthesis are shown on the left. *B. Brucella* trafficking in S2 cells. Double label immunofluorescence microscopy of: 1. *Brucella* (16M, red) (at 6 h.p.i.) and the late endosome marker mannose 6-phosphate receptor (M6PR, green); 2. *Brucella*-GFP (at 24 h.p.i) and the lysosomal marker cathepsin D (red); 3. *Brucella* (red, at 24 h.p.i) and the GFP-tagged Golgi marker D-GRASP (green). Scale bar: 5 μm.
(8.23 MB TIF)

Figure S6 Effects of selected drugs on *Brucella* or *Drosophila* S2 host cell growth. No: No drug control, 1% ddH2O (V/V); DMSO: dimethyl sulfoxide, 1% (V/V); MT: Methanol, 1% (V/V); BAF: balifomycin A1, 200 nM; BFA: brefeldin A, 2.5 μg/ml; CD: cytochalasin D, 2.5 μg/ml; WM: wortmannin, 100 nM; MR: myriocin, 10 μM. A. The indicated drugs were added into fresh TSB and *Brucella* (S2308) was incubated in this medium for the indicated periods of time. The effects of the drugs on *Brucella* growth were determined using gentamicin protection assays. 1 hr (white bars) and 72 hr (gray bars) represents the relative amount of *Brucella* in the drug-treated media (CFU/ml) compared with the untreated control at 1 and 72 hrs post coincubation, respectively. Replication efficiency (black bars) indicates relative *Brucella* replication efficiency in drug-treated medium and no drug treated control. B. Viability of *Brucella* (S2308) infected and drug-treated S2 cells. S2 cells were pretreated with drugs for 1 hr, and then infected with bacteria. At 72 h.p.i., cells were stained with trypan blue, fixed, and the percentage of viable cells was determined (Panel C). Two images, containing a total of at least 500 cells in each sample, were analyzed in each experiment. C. Images of infected S2 cells coincubated with the indicated drugs at 72 h.p.i., (1) BAF (100 nM), (2) BFA, (3) CD, (4) WM, (5) MR, and (6) No drug control. Data represent the means ± standard deviations from three independent experiments. The images were taken from a representative experiment.
(0.49 MB PDF)

Figure S7 Effect of selected drugs on *Brucella* entry and replication. A. *Drosophila* S2 cells were conincubated with assorted drugs at the indicated concentrations 1 hr before and during infection with *B. abortus* S2308 at an MOI of 100. The infected cells were lysed after 1.5 hr (entry) or 72 hr (replication) of incubation at 29°C in *Drosophila*-SFM supplemented with 40 μg/ml gentamicin and the indicated concentrations of drugs. ** and *** indicates significance at P<0.01 and at P<0.001, respectively. BAF, BFA, MR, and WM indicate treatment of S2 cells with baliformycin A1, brefeldin A, myriocin and wortmannin at the indicated concentrations, respectively. B. Relative CFUs (% of control) for *Brucella* (S2308) infection of BAF treated S2 cells at 72 h.p.i. *Brucella* infected cells were treated with BAF at 2 h.p.i.. C. Pretreatment of S2 cells with the indicated concentration of WM has no effect on internalized *Brucella* replication efficiency. Data represent the means ± standard deviations from at least three independent experiments.
(0.75 MB PDF)

Figure S8 RNAi-mediated knockdown of *Drosophila* S2 cell gene expression alters *Brucella abortus* entry and replication. Depletion of known *Brucella* host factors disrupts BA114 (S2308*virB10*::Tn5, A) and CA180 (S2308*manBA*::Tn5, C) entry but not replication (B and D). Data represent the means ± standard deviations from three independent experiments. *** represents significant at P<0.001 compared with no RNAi control.
(1.35 MB TIF)

## References

[1] Gibbs EP (2005). Emerging zoonotic epidemics in the interconnected global community.. Vet Rec.

[2] Sarinas PS, Chitkara RK (2003). Brucellosis.. Semin Respir Infect.

[3] Godfroid J, Cloeckaert A, Liautard J-P, Kohler S, Fretin D (2005). From the discovery of the Malta fever's agent to the discovery of a marine mammal reservoir, brucellosis has continuously been a re-emerging zoonosis.. Vet Res.

[4] Sauret JM, Vilissova N (2002). Human brucellosis.. J Am Board Fam Pract.

[5] Robinson-Dunn B (2002). The microbiology laboratory's role in response to bioterrorism.. Arch Pathol Lab Med.

[6] Jimenez de Bagues MP, Dudal S, Dornand J, Gross A (2005). Cellular bioterrorism: how *Brucella* corrupts macrophage physiology to promote invasion and proliferation.. Clin Immunol.

[7] Celli J, de Chastellier C, Franchini DM, Pizarro-Cerda J, Moreno E (2003). *Brucella* evades macrophage killing via virB-dependent sustained interactions with the endoplasmic reticulum.. J Exp Med.

[8] Celli J, Salcedo SP, Gorvel JP (2005). *Brucella* coopts the small GTPase Sar1 for intracellular replication.. Proc Natl Acad Sci U S A.

[9] Sun Y-H, den Hartigh AB, Santos RL, Adams LG, Tsolis RM (2002). *virB*-Mediated survival of *Brucella* abortus in mice and macrophages is independent of a functional inducible nitric oxide synthase or NADPH oxidase in macrophages.. Infect Immun.

[10] den Hartigh AB, Sun YH, Sondervan D, Heuvelmans N, Reinders MO (2004). Differential requirements for VirB1 and VirB2 during *Brucella abortus* infection.. Infect Immun.

[11] Celli J, Gorvel JP (2004). Organelle robbery: *Brucella* interactions with the endoplasmic reticulum.. Curr Opin Microbiol.

[12] Pizarro-Cerda' J, Me'Resse S, Parton RG, Van der Goot G, Sola-Landa A (1998). *Brucella abortus* transits through the autophagic pathway and replicates in the endoplasmic reticulum of nonprofessional phagocytes.. Infect Immun.

[13] Bellaire BH, Roop RM II, Cardelli JA (2005). Opsonized virulent *Brucella abortus* replicates within nonacidic, endoplasmic reticulum-negative, LAMP-1-positive phagosomes in human monocytes.. Infect Immun.

[14] Arellano-Reynoso B, Díaz-Aparicio E, Leal-Hernández M, Hernández L, Gorvel JP (2004). Intracellular trafficking study of a RB51 *B. abortus* vaccinal strain isolated from cow milk.. Vet Microbiol.

[15] Lapaque N, Moriyon I, Moreno E, Gorvel JP (2005). *Brucella* lipopolysaccharide acts as a virulence factor.. Curr Opin Microbiol.

[16] Rittig MG, Kaufmann A, Robins A, Shaw B, Sprenger H (2003). Smooth and rough lipopolysaccharide phenotypes of *Brucella* induce different intracellular trafficking and cytokine/chemokine release in human monocytes.. J Leukoc Biol.

[17] Monreal D, Grilló MJ, González D, Marín CM, De Miguel MJ (2003). Characterization of *Brucella abortus* O-polysaccharide and core lipopolysaccharide mutants and demonstration that a complete core is required for rough vaccines to be efficient against *Brucella abortus* and *Brucella ovis* in the mouse model.. Infect Immun.

[18] Pei J, Ficht TA (2004). *Brucella abortus* rough mutants are cytopathic for macrophages in culture.. Infect Immun.

[19] Pei J, Turse JE, Wu Q, Ficht TA (2006). *Brucella abortus* rough mutants induce macrophage oncosis that requires bacterial protein synthesis and direct interaction with the macrophage.. Infect Immun.

[20] O'Callaghan D, Cazevieille C, Allardet-Servent A, Boschiroli ML, Bourg G (1999). A homologue of the *Agrobacterium tumefaciens* VirB and *Bordetella pertussis* Ptl type IV secretion systems is essential for intracellular survival of *Brucella* suis.. Mol Microbiol.

[21] Sieira R, Comerci DJ, Sánchez DO, Ugalde RA (2000). A homologue of an operon required for DNA transfer in *Agrobacterium* is required in *Brucella abortus* for virulence and intracellular multiplication.. J Bacteriol.

[22] Rola'n HG, Tsolis RM (2007). Mice lacking components of adaptive immunity show increased *Brucella abortus virB* mutant colonization.. Infect Immun.

[23] Guzmán-Verri C, Chaves-Olarte E, von Eichel-Streiber C, Lopez-Goni I, Thelestam M (2001). GTPases of the Rho subfamily are required for *Brucella abortus* internalization in nonprofessional phagocytes.. J Biol Chem.

[24] Kim T, Kim YJ (2005). Overview of innate immunity in *Drosophila.*. J Biochem Mol Biol.

[25] Rämet M, Manfruelli P, Pearson A, Mathey-Prevot B, Ezekowitz RA (2002). Functional genomic analysis of phagocytosis and identification of a *Drosophila* receptor for *E. coli*.. Nature.

[26] Agaisse H, Burrack LS, Philips JA, Rubin EJ, Perrimon N (2005). Genome-wide RNAi screen for host factors required for intracellular bacterial infection.. Science.

[27] Cheng LW, Viala JP, Stuurman N, Wiedemann U, Vale RD (2005). Use of RNA interference in *Drosophila* S2 cells to identify host pathways controlling compartmentalization of an intracellular pathogen.. Proc Natl Acad Sci U S A.

[28] Philips JA, Rubin EJ, Perrimon N (2005). *Drosophila* RNAi screen reveals CD36 family member required for mycobacterial infection.. Science.

[29] Dorer MS, Kirton D, Bader JS, Isberg RR (2006). RNA interference analysis of *Legionella* in *Drosophila* cells: exploitation of early secretory apparatus dynamics.. PLoS Pathog.

[30] Derré I, Pypaert M, Dautry-Varsat A, Agaisse H (2007). RNAi screen in *Drosophila* cells reveals the involvement of the Tom complex in *Chlamydia* infection.. PLoS Pathog.

[31] Elwell CA, Ceesay A, Kim JH, Kalman D, Engel JN (2008). RNA interference screen identifies Abl kinase and PDGFR signaling in *Chlamydia trachomatis* entry.. PLoS Pathog.

[32] Lin JH, Li H, Yasumura D, Cohen HR, Zhang C (2007). IRE1 signaling affects cell fate during the unfolded protein response.. Science.

[33] Schröder M, Kohno K (2007). Recent advances in understanding the unfolded protein response.. Antioxid Redox Signal.

[34] Kohno K (2007). How transmembrane proteins sense endoplasmic reticulum stress.. Antioxid Redox Signal.

[35] Wu Q, Pei J, Turse C, Ficht TA (2006). Mariner mutagenesis of *Brucella melitensis* reveals genes with previously uncharacterized roles in virulence and survival.. BMC Microbiol.

[36] Kahl-McDonagh MM, Ficht TA (2006). Evaluation of protection afforded by *Brucella abortus* and *Brucella melitensis* unmarked deletion mutants exhibiting different rates of clearance in BALB/c mice.. Infect Immun.

[37] Tsruya R, Schlesinger A, Reich A, Gabay L, Sapir A (2002). Intracellular trafficking by Star regulates cleavage of the *Drosophila* EGF receptor ligand Spitz.. Genes & Dev.

[38] Kondylis V, Spoorendonk KM, Rabouille C (2005). dGRASP localization and function in the early exocytic pathway in *Drosophila* S2 cells.. Mol Biol Cell.

[39] Porte F, Liautard JP, Köhler S (1999). Early acidification of phagosomes containing *Brucella suis* is essential for intracellular survival in murine macrophages.. Infect Immun.

[40] Pei J, Turse JE, Ficht TA (2008). Evidence of *Brucella abortus* OPS dictating uptake and restricting NF-κB activation in murine macrophages.. Microb Infect.

[41] Miyake Y, Kozutsumi Y, Nakamura S, Fujita T, Kawasaki T (1995). Serine palmitoyltransferase is the primary target of a sphingosine-like immunosuppressant, ISP-1/myriocin. Biochem.. Biophys Res Commun.

[42] Porte F, Naroeni A, Ouahrani-Bettache S, Liautard JP (2003). Role of the *Brucella suis* lipopolysaccharide O antigen in phagosomal genesis and in inhibition of phagosome-lysosome fusion in murine macrophages.. Infect Immun.

[43] Brachmann SM, Yballe CM, Innocenti M, Deane JA, Fruman DA (2005). Role of phosphoinositide 3-kinase regulatory isoforms in development and actin rearrangement.. Mol Cell Biol.

[44] Kondo S, Murakami T, Tatsumi K, Ogata M, Kanemoto S, Otori K, Iseki K, Wanaka A, Imaizumi K (2005). OASIS, a CREB/ATF-family member, modulates UPR signalling in astrocytes.. Nat Cell Biol.

[45] Kondo S, Saito A, Hino S-i, Murakami T, Ogata M (2007). BBF2H7, a novel transmembrane bZIP transcription factor, is a new type of endoplasmic reticulum stress transducer.. Mol Cell Biol.

[46] Zhang K, Kaufman RJ (2006). The unfolded protein response: a stress signaling pathway critical for health and disease.. Neurology.

[47] Naroeni A, Porte F (2002). Role of cholesterol and the ganglioside GM(1) in entry and short-term survival of *Brucella suis* in murine macrophages.. Infect Immun.

[48] Watarai M, Makino S, Fujii Y, Okamoto K, Shirahata T (2002). Modulation of *Brucella*-induced macropinocytosis by lipid rafts mediates intracellular replication.. Cell Microbiol.

[49] Grassmé H, Jendrossek V, Riehle A, von Kürthy G, Berger J (2003). Host defense against *P. aeruginosa* requires ceramide-rich membrane rafts.. Nat Med.

[50] Gulbins E, Dreschers S, Wilker B, Grassmé H (2004). Ceramide, membrane rafts and infections.. J Mol Med.

[51] Köhler S, Teyssier J, Cloeckaert A, Rouot B, Liautard JP (1996). Participation of the molecular chaperone DnaK in intracellular growth of *Brucella suis* within U937-derived phagocytes.. Mol Microbiol.

[52] Cheng LW, Portnoy DA (2003). *Drosophila* S2 cells: an alternative infection model for *Listeria monocytogenes*.. Cell Microbiol.

[53] Salcedo SP, Holden DW (2005). Bacterial interactions with the eukaryotic secretory pathway.. Current Opinion in Microbiology,.

[54] Roy CR, Salcedo SP, Gorvel JP (2006). Pathogen-endoplasmic-reticulum interactions: in through the out door.. Nat Rev Immunol 2006.

[55] Friedlander R, Jarosch E, Urban J, Volkwein C, Sommer T (2000). A regulatory link between ER-associated protein degradation and the unfolded-protein response.. Nat Cell Biol,.

[56] Kincaid MM, Cooper AA (2007). Eradicate ER stress or die trying.. Antioxidants & Redox Signaling.

[57] Chang HJ, Jesch SA, Gaspar ML, Henry SA (2004). Role of the unfolded protein response pathway in secretory stress and regulation of *INO1* expression in *Saccharomyces cerevisiae*.. Genetics.

[58] Stroobants AK, Hettema EH, van den Berg M, Tabak HF (1999). Enlargement of the endoplasmic reticulum membrane in *Saccharomyces cerevisiae* is not necessarily linked to the unfolded protein response *via* Ire1p.. FEBS Lett.

[59] Bernales S, McDonald KL, Walter P (2006). Autophagy counterbalances endoplasmic reticulum expansion during the unfolded protein response.. PLoS Biol.

[60] Ogata M, Hino S, Saito A, Morikawa K, Kondo S (2006). Autophagy is activated for cell survival after endoplasmic reticulum stress.. Mol Cell Biol.

[61] Ishihara N, Hamasaki M, Yokota S, Suzuki K, Kamada Y (2001). Autophagosome requires specific early Sec proteins for its formation and NSF/SNARE for vacuolar fusion.. Mol Biol Cell.

[62] Hamasaki M, Noda T, Ohsumi Y (2003). The early secretory pathway contributes to autophagy in yeast.. Cell Struct Funct.

[63] Reggiori F, Wang CW, Nair U, Shintani T, Abeliovich H (2004). Early stages of the secretory pathway, but not endosomes, are required for Cvt vesicle and autophagosome assembly in *Saccharomyces cerevisiae*.. Mol Biol Cell.

[64] Vieira OV, Botelho RJ, Rameh L, Brachmann SM, Matsuo T (2001). Distinct roles of class I and class III phosphatidylinositol 3-kinases in phagosome formation and maturation.. J Cell Biol.

[65] Vieira OV, Bucci C, Harrison RE, Trimble WS, Lanzetti L (2003). Modulation of Rab5 and Rab7 recruitment to phagosomes by phosphatidylinositol 3-kinase.. Mol Cell Biol.

[66] Tassa A, Roux MP, Attaix D, Bechet DM (2003). Class III phosphoinositide 3-kinase–Beclin1 complex mediates the amino acid-dependent regulation of autophagy in C2C12 myotubes.. Biochem J.

[67] Lee K, Tirasophon W, Shen X, Michalak M, Prywes R (2002). IRE1-mediated unconventional mRNA splicing and S2P-mediated ATF6 cleavage merge to regulate XBP1 in signaling the unfolded protein response.. Genes & Dev.

[68] Blais JD, Addison CL, Edge R, Falls T, Zhao H (2006). Perk-dependent translational regulation promotes tumor cell adaptation and angiogenesis in response to hypoxic stress.. Mol Cell Biol.

