# Supplementary material for: RNAi Screen of Endoplasmic Reticulum–Associated Host Factors Reveals a Role for IRE1α in Supporting Brucella Replication
Source: PLoS Pathog. 2008 Jul 25;4(7):e1000110. doi: 10.1371/journal.ppat.1000110 (PMC2453327; doi:10.1371/journal.ppat.1000110)
Supplement: Table S1 — Brucella strains used in this study (0.14 MB DOC) [file ppat.1000110.s001.doc]

Table S1. *Brucella* strains used in this study

| Species/strain | Characteristics of the strains | Reference |
| --- | --- | --- |
| *Brucella abortus* |  |  |
| S2308 | Wild type strain | [[1](#Pei), [2](#Kahl-McDonagh_)] |
| S19 | Vaccine strain | [[2](#Kahl-McDonagh_)] |
| RB51 | Vaccine strain | [[2](#Kahl-McDonagh_)] |
| BA114 | S2308*virB10*::Tn5 | [[3](#Hong)] |
| CA180 | S2308*manB*::Tn5 | [[1](#Pei), [4](#Allen)] |
| S2308*∆manBA* | *manBA::*Km, derived from S2308 | [[2](#Kahl-McDonagh_)] |
| S2308*∆virB2* | *ΔvirB2,* derived from S2308 | [[2](#Kahl-McDonagh_)] |
| *B. melitensis* |  |  |
| 16M | Wild type strain | [[2](#Kahl-McDonagh_)] |
| 16M-GFP | GFP expressing strain, 16M-pBBR1MCS-6Y | Weeks et al. ^a^ |
| 16MΔ*manBA* | ∆*manBA*, derived from 16M | [[2](#Kahl-McDonagh_)] |
| 16MΔ*virB2* | ∆*virB2*, derived from 16M | [[2](#Kahl-McDonagh_)] |
| 102B2 (BMEI1364) | 16M*mucR*::Himar1, derived from 16M | [[5](#Wu)] |
| 146D5 (BMEI1178) | 16M*merR*::Himar1, derived from 16M | [[5](#Wu)] |

**^a^** Unpublished data

1. Pei J, and Ficht TA (2004) *Brucella* abortus rough mutants are cytopathic for macrophages in culture*.* Infect Immun 72: 440-450.
2. Kahl-McDonagh MM, Ficht TA (2006) Evaluation of protection afforded by *Brucella abortus* and *Brucella melitensis* unmarked deletion mutants exhibiting different rates of clearance in BALB/c mice. Infect Immun 74:4048-4057.
3. Hong PC, Tsolis RM, and Ficht TA (2000) Identification of Genes Required for Chronic Persistence of *Brucella abortus* in Mice. Infect Immun 68: 4102–4107.
4. Hong CA, Adams LG, and Ficht TA (1998) Transposon-derived *Brucella abortus* rough mutants are attenuated and exhibit reduced intracellular survival. Infect Immun 66:1008-1016.
5. Wu Q, [Pei J](http://www.ncbi.nlm.nih.gov/sites/entrez?Db=pubmed&Cmd=Search&Term="Pei J"%5BAuthor%5D&itool=EntrezSystem2.PEntrez.Pubmed.Pubmed_ResultsPanel.Pubmed_RVAbstractPlus), [Turse C](http://www.ncbi.nlm.nih.gov/sites/entrez?Db=pubmed&Cmd=Search&Term="Turse C"%5BAuthor%5D&itool=EntrezSystem2.PEntrez.Pubmed.Pubmed_ResultsPanel.Pubmed_RVAbstractPlus), [Ficht TA](http://www.ncbi.nlm.nih.gov/sites/entrez?Db=pubmed&Cmd=Search&Term="Ficht TA"%5BAuthor%5D&itool=EntrezSystem2.PEntrez.Pubmed.Pubmed_ResultsPanel.Pubmed_RVAbstractPlus) (2006) Mariner mutagenesis of *Brucella melitensis* reveals genes with previously uncharacterized roles in virulence and survival*.* BMC Microbiol 6: 102.
