# Supplementary material for: RNAi Screen of Endoplasmic Reticulum–Associated Host Factors Reveals a Role for IRE1α in Supporting Brucella Replication
Source: PLoS Pathog. 2008 Jul 25;4(7):e1000110. doi: 10.1371/journal.ppat.1000110 (PMC2453327; doi:10.1371/journal.ppat.1000110)
Supplement: Table S2 — Comparison of Drosophila S2 and mammalian cells treated with drugs that inhibit Brucella abortus (S2308) entry and replication (0.07 MB DOC) [file ppat.1000110.s002.doc]

Table S2. Comparison of *Drosophila* S2 and mammalian cells treated with drugs that inhibit *Brucella abortus* (S2308) entry and replication

| Drug Name | Target/Process | Concentration  (μM) | CFU (% of control) ^a^ | | |  |
| --- | --- | --- | --- | --- | --- | --- |
|  |  |  | S2 cells | | Mammalian cells | Reference |
|  |  |  | Entry | Replication | Entry |  |
| Balifomycin A1 | Vacuolar H^+^-ATPase | 0.1 | 29.1±5.5***^b^ | 30.6±2.5*** | 45.7 ^c^ | [[1](#Porte), this study] |
| Brefeldin A | Secretion endocytosis | 8.9 | 86.3±28.6 | 87.0±21.6 | NA ^d^ | [[2](#Celli), this study] |
| Cytochalasin D | Actin polymerization | 5.0 | 1.8±0.7*** | ND ^e^ | 1.2±0.4 | [[3](#Guzm), this study] |
| Myriocin | Sphingolipid biosynthesis | 10.0 | 51.2±9.2*** | 24.0±2.8*** | ND | This study |
| Wortmannin | PI3-kinases | 0.1 | 31.6±5.9*** | 34.4±6.4*** | 10.0 ^f^ | [[3](#Guzm·n-Verri_), this study] |

^a^ Colony forming units (CFU) of the untreated control [(3.1±0.4)×10^5^/well, (1.6±0.1)×10^7^/well for entry and replication, respectively] was normalized as 100%. ^b^ Data represent the means ± standard deviations from three independent experiments. *** represents significant at P<0.001 compared with the untreated control. ^c^ Result from murine macrophage J774.A1 cells infected with *B. suis* at 4 h.p.i. ^d^ Mice bone marrow derived macrophages (BMDMs) infected with wild type *B. abortus* and the treatment of brefeldin A has no effect on*Brucella* entry, replication and intracellular trafficking, and no accurate statistic data shown in the reference.  ^e^ Not detected. ^f^ HeLa cells pretreated with 50 nM Wortmannin and infection with *B. abortus*.

- - - 1. Porte F, Liautard JP, Köhler S. (1999) Early acidification of phagosomes containing Brucella suis is essential for intracellular survival in murine macrophages. Infect Immun 67: 4041-4047.
      2. Celli J, Salcedo SP, and Gorvel JP (2005) *Brucella* coopts the small GTPase Sar1 for intracellular replication. Proc Natl Acad Sci U S A 102: 1673-1678.
      3. Guzmán-Verri C, Chaves-Olarte E, von Eichel-Streiber C, Lopez-Goni I, Thelestam M, et al. (2001) GTPases of the Rho subfamily are required for *Brucella abortus* internalization in nonprofessional phagocytes. J Biol Chem 276: 44435-44443.
