# Supplementary figures and images for: RNAi Screen of Endoplasmic Reticulum–Associated Host Factors Reveals a Role for IRE1α in Supporting Brucella Replication
Source: PLoS Pathog. 2008 Jul 25;4(7):e1000110. doi: 10.1371/journal.ppat.1000110 (PMC2453327; doi:10.1371/journal.ppat.1000110)

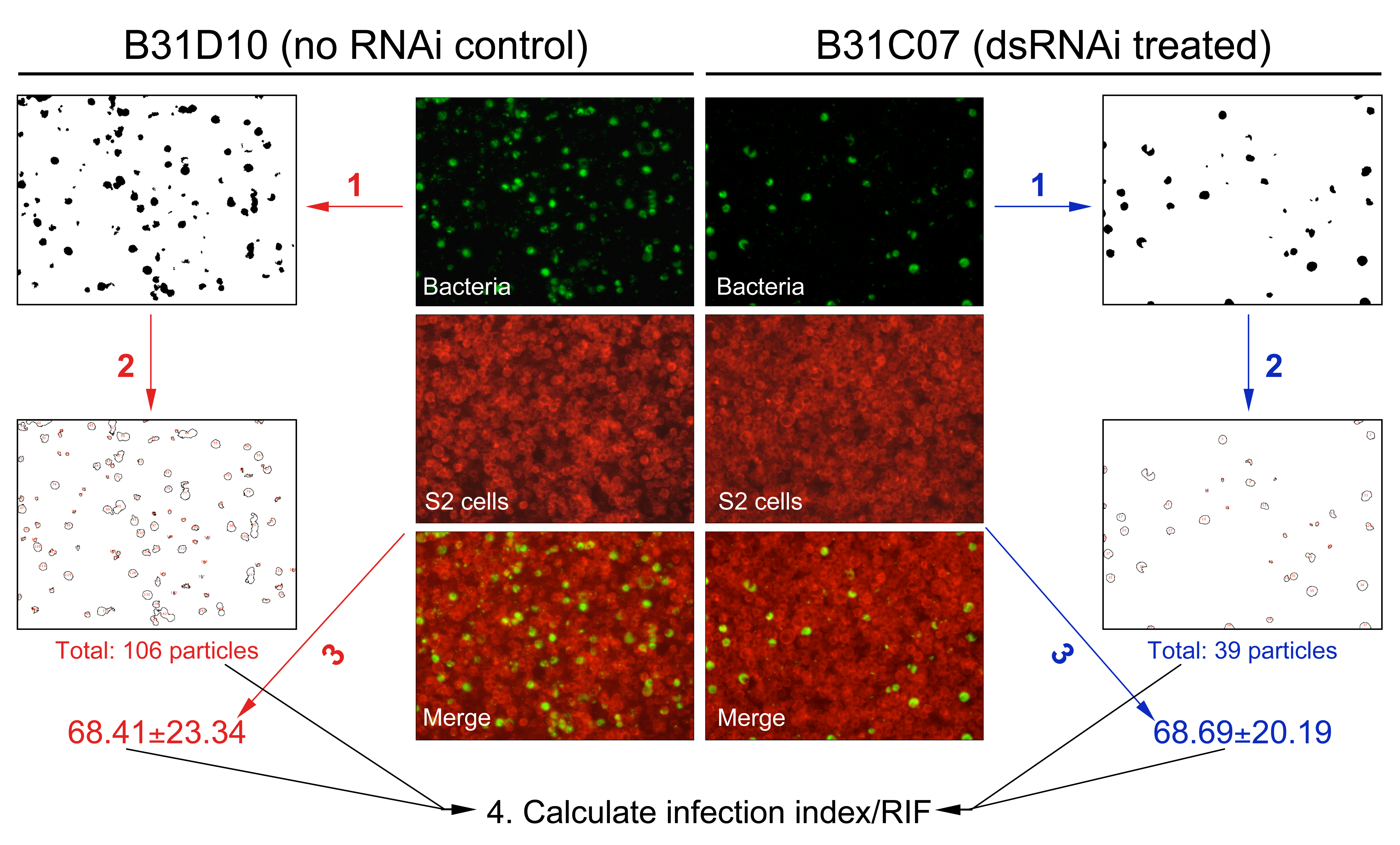

Supplement: Figure S1 — Schematic representation of image analysis using Image J to calculate the relative infection (RIF). 1. Threshold of bacterial replication in infected cells in an image using the same setting. 2. Analysis of particles in a thresholed image (i.e., the number of infected cells with bacterial replication). 3. Histogram of the image (cell numbers were adjusted via color density). 4. Calculation of the infection index and RIF (% of control) of the samples. For example, infection index of sample B31C07 = 39/68.69; RIF of B31C07 = 100×[39/68.69]/[106/68.41] = 36.12. (8.15 MB TIF) [file ppat.1000110.s004.tif]

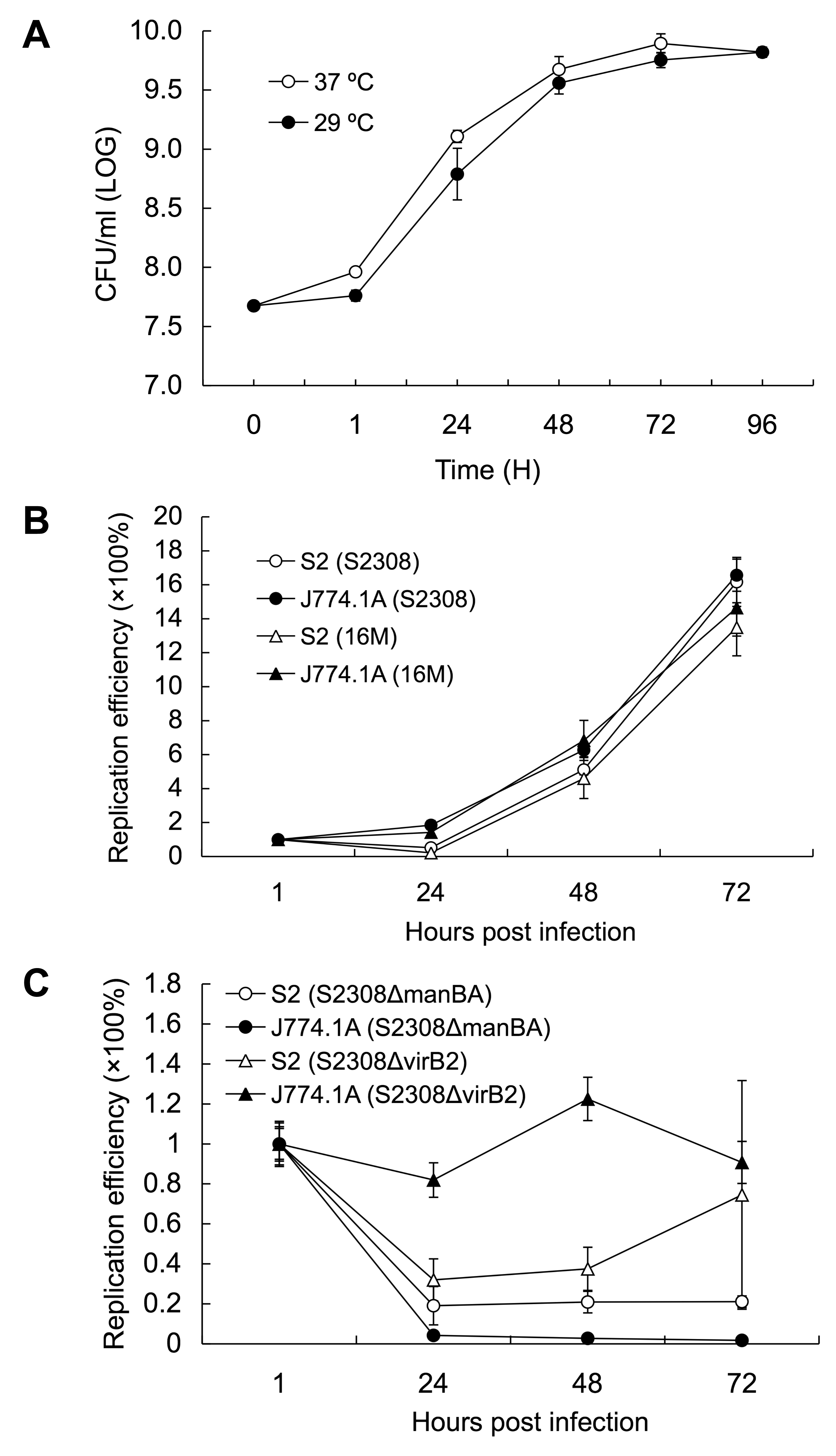

Supplement: Figure S2 — Brucella abortus and B. melitensis growth, and infection of host cells, at 29°C and 37°C. A. B. abortus growth in liquid culture (TSB) at 29°C and 37°C. B. Replication of Brucella wild-type strains S2308 and 16M in Drosophila S2 and J774.A1 murine macrophages at 29°C. The number of CFUs of 16M and S2308 for S2 [(4.18±0.54) ×105/well and (3.51±0.95) ×105/well, respectively] and for J774.A1 [(5.17±0.25) ×105/well and (7.77±0.47) ×105/well, respectively] cells at 1 h.p.i was normalized as 100%. Brucella replication efficiency was defined as the number of CFUs at different time points post infection/the number of CFUs of bacterial entry (1 h.p.i). C. Entry and replication of S2308 derived mutants in S2 and J774.A1 murine macrophages at 29°C. Data represent the means ± standard deviations from three independent experiments. (0.50 MB PDF) [file ppat.1000110.s005.tif]

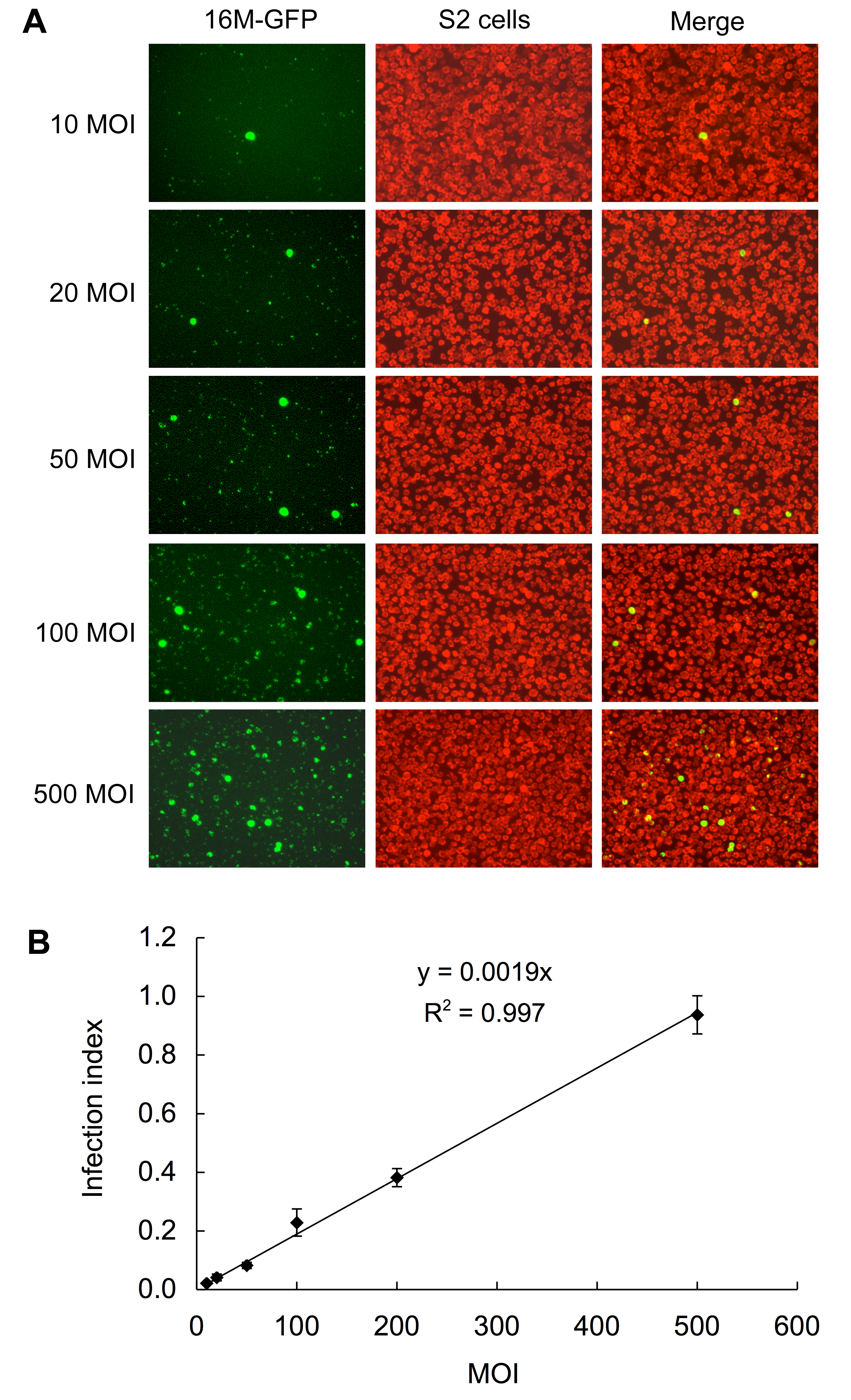

Supplement: Figure S3 — Infection of Drosophila S2 cells increases with rising multiplicity of infection (MOI). A. With increasing MOI, the number of S2 cells containing replicating Brucella melitensis (16M-GFP) increases at 72 h.p.i. The images were taken from a representative experiment. B. Infection index (i.e., Number of infected S2 cells with replicating Brucella/total cell number based on image analysis) and MOI display a linear relationship in the range of tested MOI. Data represent the means ± standard deviations from three independent experiments. (10.28 MB TIF) [file ppat.1000110.s006.tif]

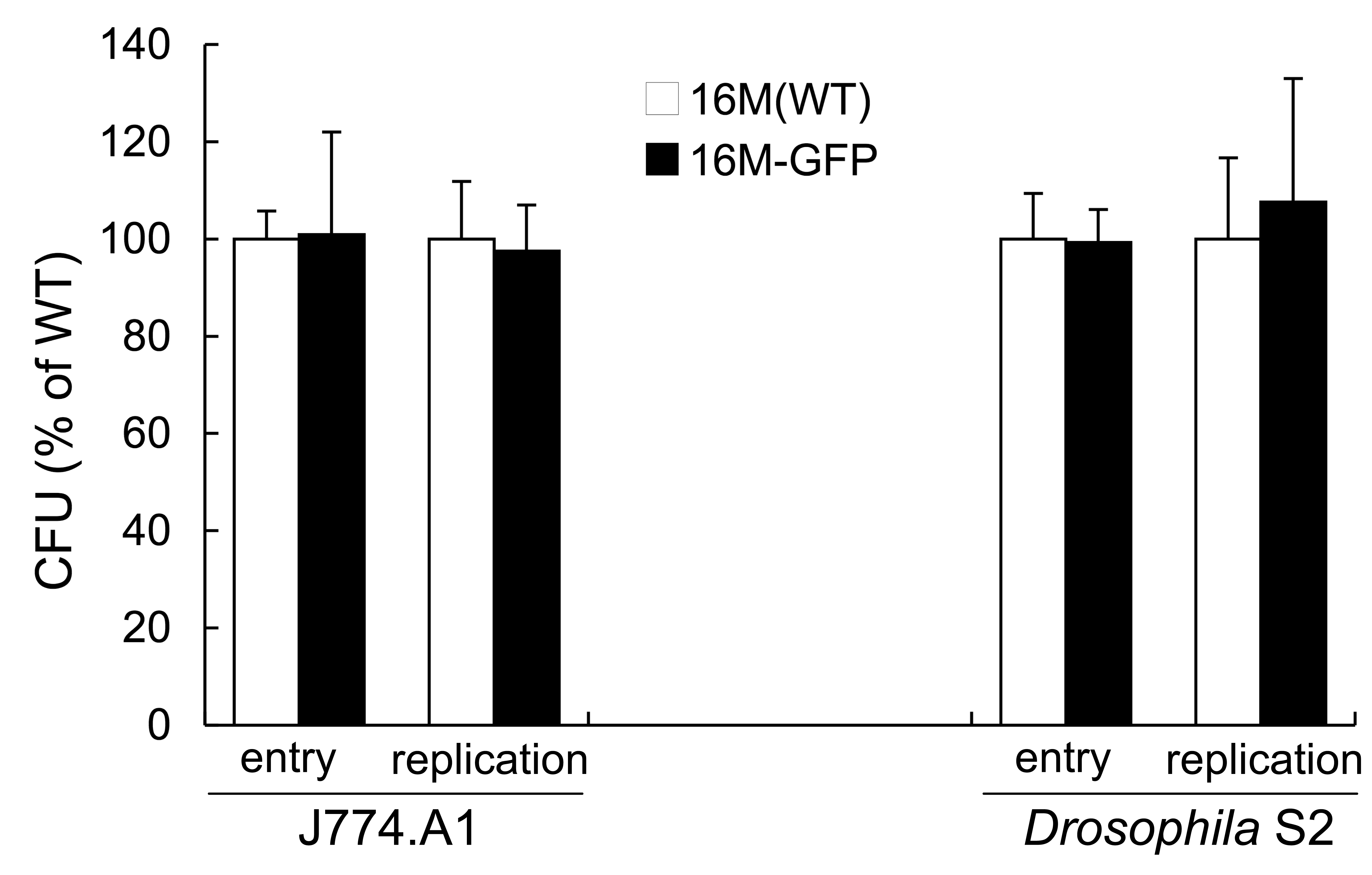

Supplement: Figure S4 — GFP expression has no effect on bacterial entry and replication. The entry (1.5 h.p.i) and replication (72 h.p.i.) of Brucella melitensis strains 16M and 16M-GFP in Drosophila S2 and J774.A1 murine macrophages at 29°C and 37°C, respectively, were compared using gentamicin protection assays. The number of 16M CFUs of entry and replication in Drosophila S2 cells [(4.85±0.46) ×105/well and (8.89±1.23) ×106/well, respectively] and in J774.A1 cells [(3.68±0.29) ×105/well and (1.77±0.21) ×107/well, respectively] were normalized as 100%. No significant differences in entry or replication in S2 or J774.A1 cells were observed in the two strains. Data represent the means ± standard deviations from three independent experiments. (0.30 MB TIF) [file ppat.1000110.s007.tif]

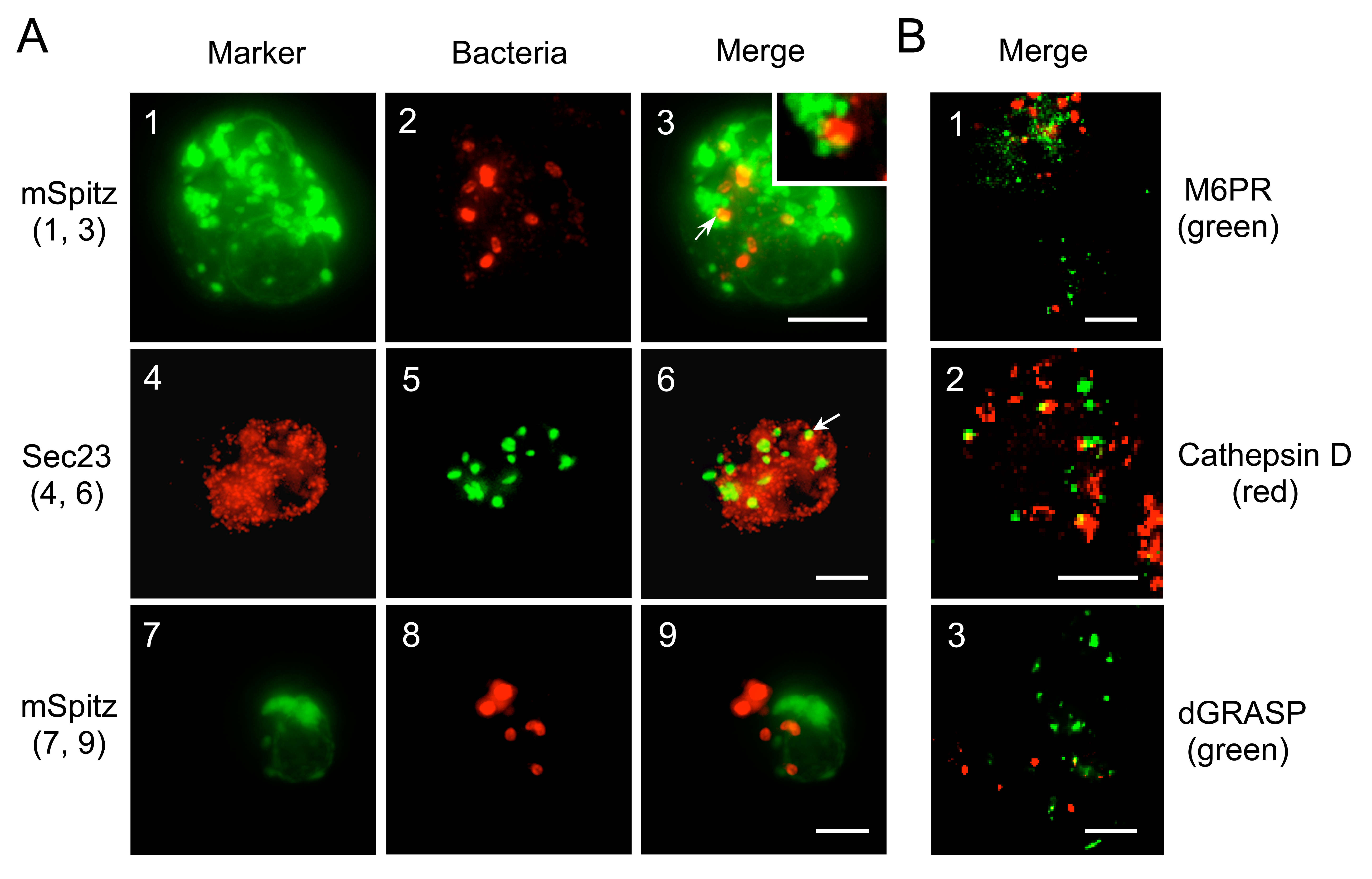

Supplement: Figure S5 — Brucella melitensis intracellular trafficking in Drosophila S2 cells. A. 1. S2 cells expressing a GFP-tagged variant of the Drosophila ER maker mSpitz (mSpitz-GFP). 2. B. melitensis (16M) infection of S2 cells (24 h.p.i). 3. A tight association between B. melitensis cells and host cell ER membranes (arrow) in the main and inset panels is observed in the merged image. 4. Immunofluorescence localization of COPII in S2 cells using Sec23 polyclonal antibodies. 5. 16M-GFP localization in S2 cells at 12 h.p.i. (Green, Panel A). 6. A merged image showing COPII proteins and B. melitensis (16M-GFP) localization. 7. mSpitz-GFP localization in S2 cells. 8. Fixed and killed B. melitensis (16M) in S2 cells at 24 h.p.i. 9. Merged panels 7 and 8. Markers used in the panels indicated in parenthesis are shown on the left. B. Brucella trafficking in S2 cells. Double label immunofluorescence microscopy of: 1. Brucella (16M, red) (at 6 h.p.i.) and the late endosome marker mannose 6-phosphate receptor (M6PR, green); 2. Brucella-GFP (at 24 h.p.i) and the lysosomal marker cathepsin D (red); 3. Brucella (red, at 24 h.p.i) and the GFP-tagged Golgi marker D-GRASP (green). Scale bar: 5 μm. (8.23 MB TIF) [file ppat.1000110.s008.tif]

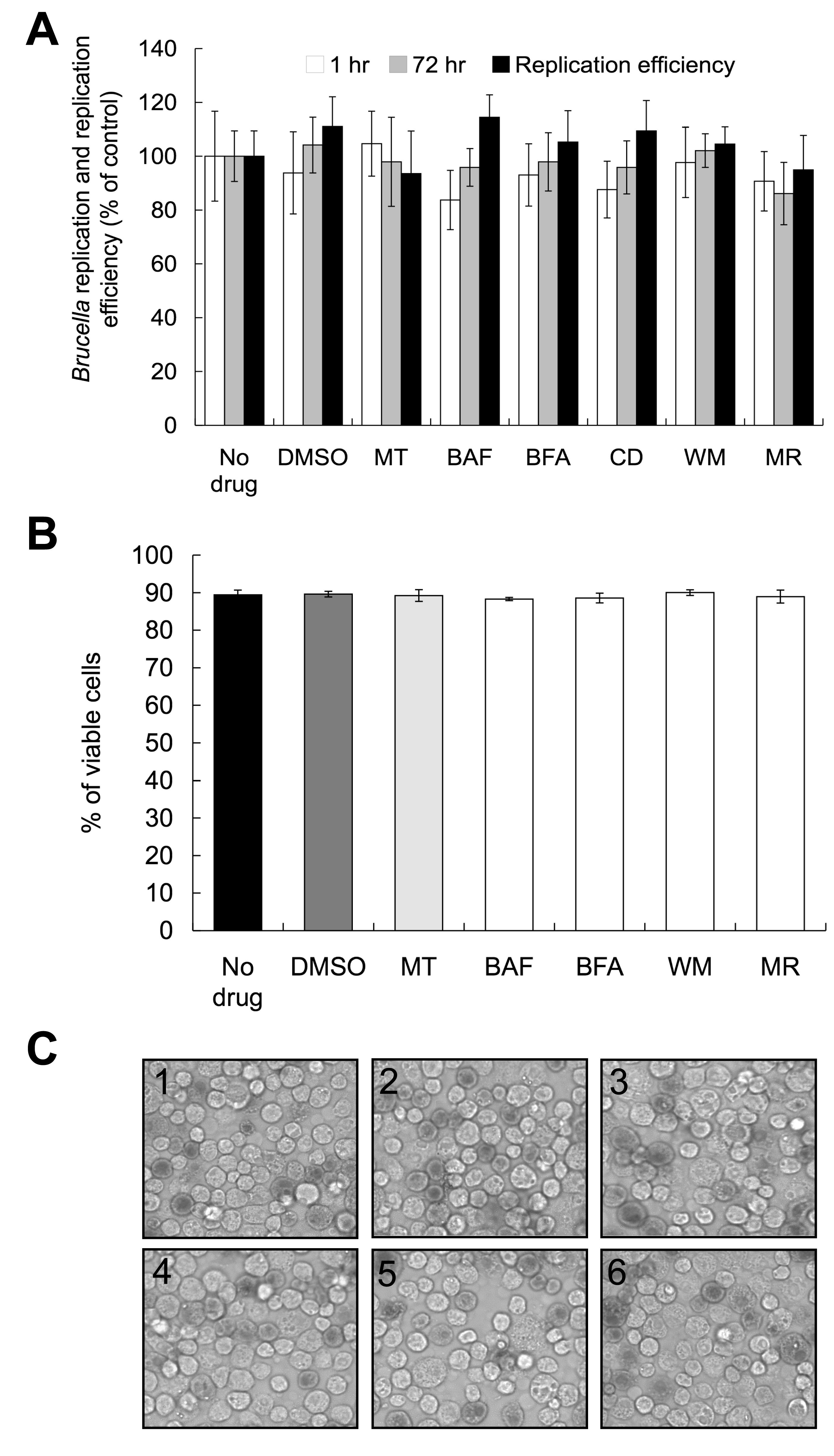

Supplement: Figure S6 — Effects of selected drugs on Brucella or Drosophila S2 host cell growth. No: No drug control, 1% ddH2O (V/V); DMSO: dimethyl sulfoxide, 1% (V/V); MT: Methanol, 1% (V/V); BAF: balifomycin A1, 200 nM; BFA: brefeldin A, 2.5 μg/ml; CD: cytochalasin D, 2.5 μg/ml; WM: wortmannin, 100 nM; MR: myriocin, 10 μM. A. The indicated drugs were added into fresh TSB and Brucella (S2308) was incubated in this medium for the indicated periods of time. The effects of the drugs on Brucella growth were determined using gentamicin protection assays. 1 hr (white bars) and 72 hr (gray bars) represents the relative amount of Brucella in the drug-treated media (CFU/ml) compared with the untreated control at 1 and 72 hrs post coincubation, respectively. Replication efficiency (black bars) indicates relative Brucella replication efficiency in drug-treated medium and no drug treated control. B. Viability of Brucella (S2308) infected and drug-treated S2 cells. S2 cells were pretreated with drugs for 1 hr, and then infected with bacteria. At 72 h.p.i., cells were stained with trypan blue, fixed, and the percentage of viable cells was determined (Panel C). Two images, containing a total of at least 500 cells in each sample, were analyzed in each experiment. C. Images of infected S2 cells coincubated with the indicated drugs at 72 h.p.i., (1) BAF (100 nM), (2) BFA, (3) CD, (4) WM, (5) MR, and (6) No drug control. Data represent the means ± standard deviations from three independent experiments. The images were taken from a representative experiment. (0.49 MB PDF) [file ppat.1000110.s009.tif]

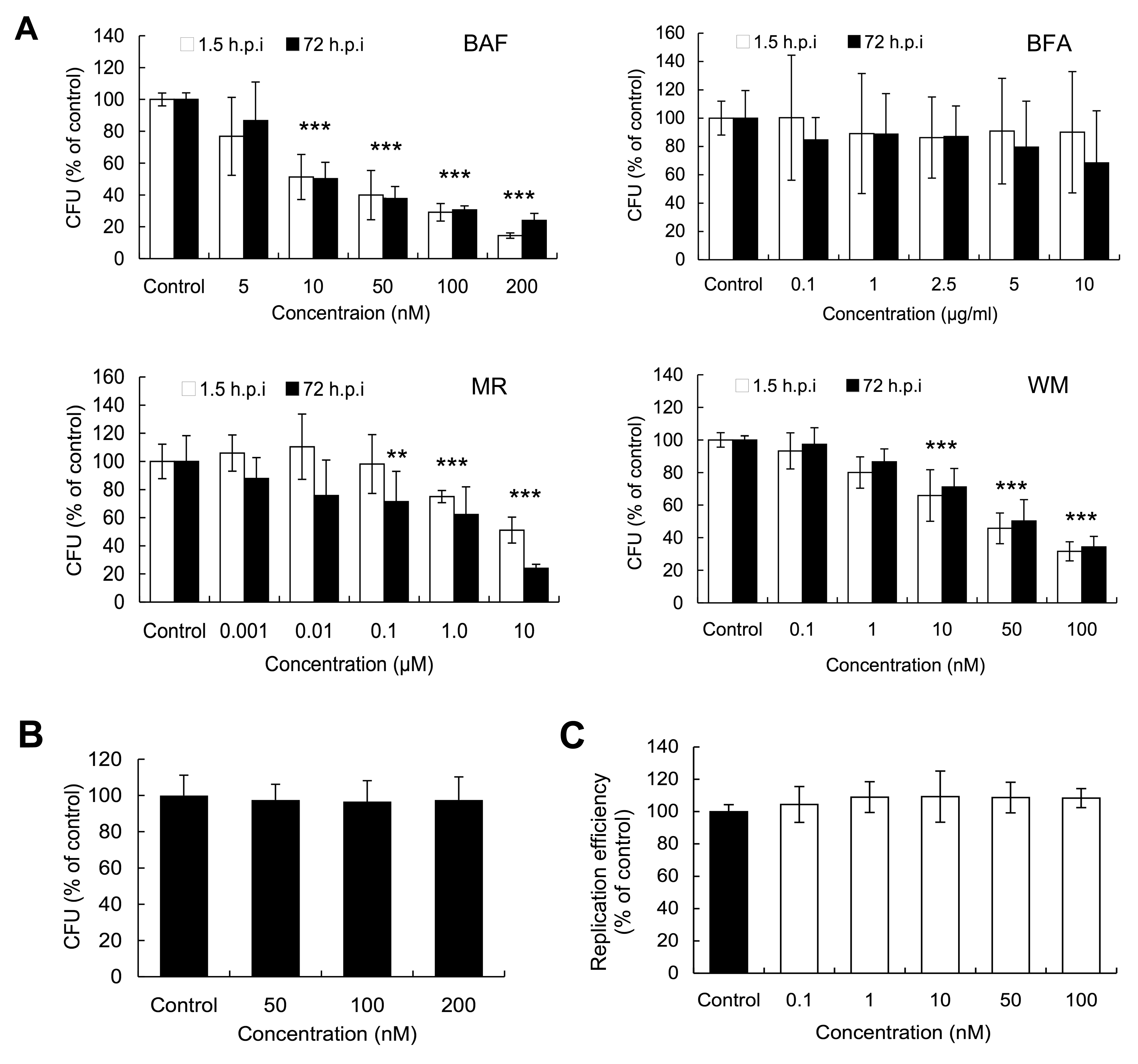

Supplement: Figure S7 — Effect of selected drugs on Brucella entry and replication. A. Drosophila S2 cells were conincubated with assorted drugs at the indicated concentrations 1 hr before and during infection with B. abortus S2308 at an MOI of 100. The infected cells were lysed after 1.5 hr (entry) or 72 hr (replication) of incubation at 29°C in Drosophila-SFM supplemented with 40 μg/ml gentamicin and the indicated concentrations of drugs. ** and *** indicates significance at P<0.01 and at P<0.001, respectively. BAF, BFA, MR, and WM indicate treatment of S2 cells with baliformycin A1, brefeldin A, myriocin and wortmannin at the indicated concentrations, respectively. B. Relative CFUs (% of control) for Brucella (S2308) infection of BAF treated S2 cells at 72 h.p.i. Brucella infected cells were treated with BAF at 2 h.p.i.. C. Pretreatment of S2 cells with the indicated concentration of WM has no effect on internalized Brucella replication efficiency. Data represent the means ± standard deviations from at least three independent experiments. (0.75 MB PDF) [file ppat.1000110.s010.tif]

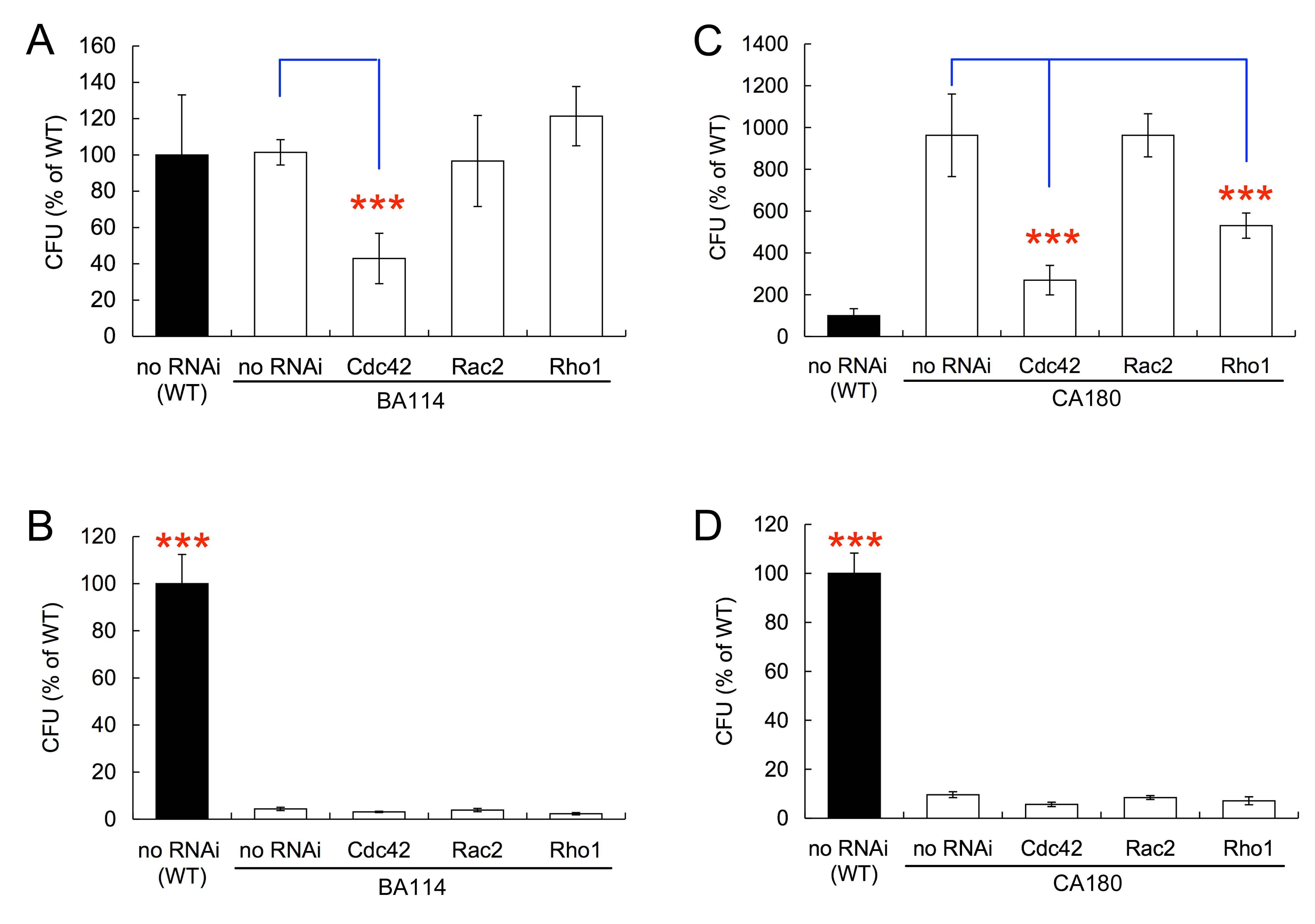

Supplement: Figure S8 — RNAi-mediated knockdown of Drosophila S2 cell gene expression alters Brucella abortus entry and replication. Depletion of known Brucella host factors disrupts BA114 (S2308virB10::Tn5, A) and CA180 (S2308manBA::Tn5, C) entry but not replication (B and D). Data represent the means ± standard deviations from three independent experiments. *** represents significant at P<0.001 compared with no RNAi control. (1.35 MB TIF) [file ppat.1000110.s011.tif]
